# M2 macrophage−derived exosomes improves secondary lymphedema through cellular mitochondrial homeostasis regulation via the Keap1-Nrf2/mPTP axis

**DOI:** 10.3389/fimmu.2026.1832261

**Published:** 2026-07-10

**Authors:** Jinli Ma, Luya Pu, Yundong Zhang, Baiao Wu, Rui Fei, Dongmei Han, Miao Hao, Jianshi Du

**Affiliations:** 1 China-Japan Union Hospital of Jilin University, Changchun, China; 2 Jilin Provincial International Joint Research Center for Lymphatic Vascular Disease, Key Laboratory of Lymphatic Surgery of Jilin Province, Changchun, China; 3 Engineering Laboratory of Lymphatic Surgery of Jilin Province, Changchun, China

**Keywords:** exosome, macrophage polarization, mitochondrial permeability transition pore, Nrf2, secondary lymphedema

## Abstract

**Background:**

Secondary lymphedema leads to progressive tissue remodeling and immune dysregulation. Dysregulation of macrophage polarization critically influences pathological progression. Oxidative stress and mitochondrial homeostasis are the critical components of secondary lymphedema.

**Methods:**

A secondary lymphedema model and an LPS-induced *in vitro* cell injury model were established. Mitochondrial dynamics were evaluated using the ROS assay, mitochondrial membrane potential analysis and mPTP opening assays. Mechanistic investigations included Nrf2 nuclear translocation analysis via immunofluorescence, western blotting, and pharmacological inhibition of exosome biogenesis using GW4869.

**Results:**

Secondary lymphedema tissues exhibited a significant imbalance in M1/M2 macrophage infiltration (with an increase in M1 and decrease in M2), along with elevated IL-1β levels. M2-Exo significantly prevents the proliferation, migration, and tube formation of human lymphatic endothelial cells and alleviated the mitochondrial damage induced by LPS. This mechanism may involve activation of Keap1-Nrf2 signaling.

**Conclusion:**

M2 macrophage exosomes activated the Nrf2 anti-oxidative stress pathway. This activation improves mitochondrial homeostasis and enhances the function of human lymphatic endothelial cells by delivering active ingredients, offering a new strategy for the treatment of secondary lymphedema.

## 1 Introduction

Secondary lymphedema (SL) is a progressive disease caused by structural damage or dysfunction of the lymphatic vessels (1, 2). The clinical etiology primarily involves iatrogenic interventions, such as radical tumor surgery, radiation therapy, infection, and trauma (3). The pathological features are primarily characterized by interstitial edema, chronic inflammation, skin fibrosis, and adipose tissue deposition (4, 5). Epidemiological studies have revealed a significant risk of upper extremity SL among patients with breast cancer. Specifically, up to 42.2% of patients receiving conventional radiotherapy may be affected (6). In patients with genitourinary and gynecological malignant tumors, up to 31% may develop lower extremity SL following radiotherapy and pelvic lymph node dissection (7). This disease severely impairs patients’ long-term quality of life, imposing both physical and psychological burdens (8, 9). However, currently used clinical approaches, such as complex decongestive therapy and surgical interventions, cannot reverse the core pathology of impaired lymphatic regeneration, highlighting the urgent need for innovative therapeutic strategies targeting the biological regulatory mechanisms of lymphatic vessels (10–12).

The pathogenesis of SL is complex and involves multi-pathway interactions, in which the vicious cycle of immune inflammation and oxidative stress is the central link driving SL progression (13–16). At the level of immune regulation, increased proportions of M1 macrophages and decreased proportions of M2 macrophages in SL tissues are considered key features of microenvironmental disturbance (17, 18). Regarding oxidative stress, SL induces excessive oxidative stress, leading to increased production of reactive oxygen species (ROS), exacerbating oxidative DNA damage in human lymphatic endothelial cells (hLECs), and affecting lymphatic function through vascular endothelial growth factor C (VEGF−C); these are important mechanisms underlying SL evolution (16, 19). Antioxidant therapy promotes the formation of new lymphatic vessels and improves lymphatic drainage efficiency, thus becoming a key approach for SL treatment (14, 20). Recent studies have shown that M2 macrophage−derived conditioned medium (M2−CM), which contains exosomes and various cytokines, plays roles in promoting tissue repair and immune regulation in ischemia−reperfusion injury, peripheral circulatory disorders, and skin regeneration (21–23). Notably, depletion of M2 macrophages reduces the number of LYVE−1−positive lymphatic vessels and exacerbates edema development (24), whereas increasing M2 macrophages promotes normal lymphatic vessel generation and ameliorates SL progression by remodeling the inflammatory microenvironment, suggesting the therapeutic potential of M2 macrophages in SL (18, 25). However, the specific roles and underlying mechanisms of M2 macrophage−derived exosomes(M2-Exo) in the treatment of SL remain unclear. Therefore, elucidating the therapeutic effects and molecular mechanisms of M2-Exo in SL holds significant clinical translational value.

This study focused on the molecular mechanisms of M2-Exo in the treatment of SL, with the aim of providing a scientific basis for SL treatment strategies. By exploring the imbalance of the immune microenvironment and the key pathway of oxidative stress in the pathogenesis of SL, this study investigated the potential role and mechanism of M2-Exo in mitigating oxidative stress-induced injury and protecting the function of hLECs. This work enriches our knowledge of the roles and mechanisms of paracrine effect of M2 macrophages on the regulation of mitochondrial homeostasis in hLECs. Furthermore, it provides theoretical support for novel SL treatment strategies based on the immune microenvironment by revealing the regulatory network of M2-Exo in the dynamic balance of mPTP.

## 2 Methods

### 2.1 Mouse-tail model of secondary lymphedema

**Figure 1:**
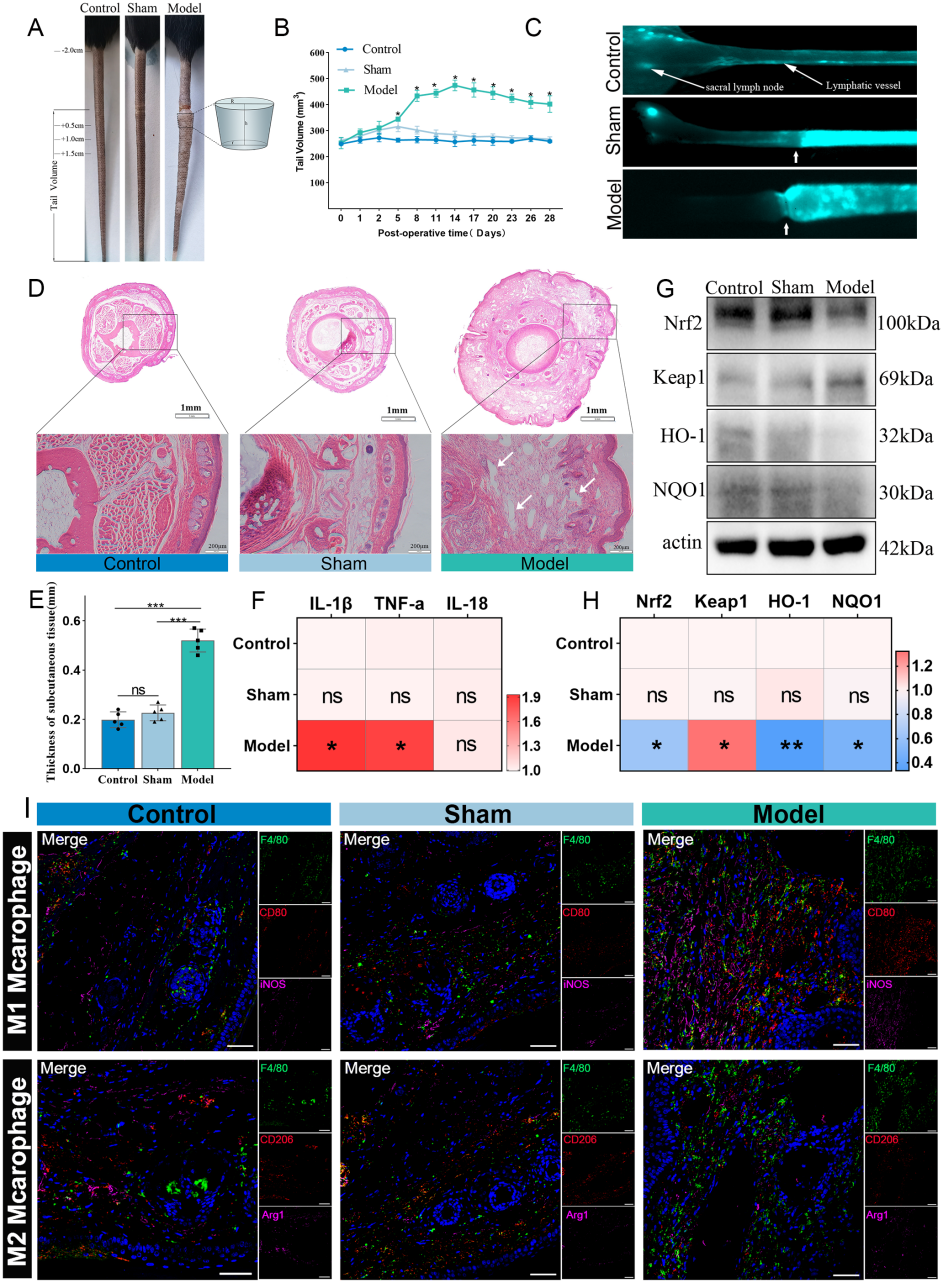
Reduced M2 macrophage infiltration in tissues of secondary lymphedema. **(A)** Photographs of mouse tail at 4 weeks after lymphatic surgery; **(B)** Line graph of volume change of mouse tail; **(C)** NIR-II imaging of mouse tail at 4 weeks after lymphatic surgery; **(D)** H&E staining of mouse tail subcutaneous tissue at 4 weeks after lymphatic surgery (white arrows indicate dilated lymphatic vessels); **(E)** Thickness of subcutaneous tissues of the tail at 4 weeks after lymphatic surgery (μm); **(F)** Relative expression of inflammatory factor mRNAs in the tail tissues of the mouse at 4 weeks after lymphatic surgery; **(G)** Representative WB images in the tail tissues at 4 weeks after lymphatic surgery; **(H)** Quantitative analysis of Nrf2/Keap1/HO-1/NQO1 expression; **(I)** Multiplex immunofluorescence staining of macrophage in mouse tail tissues 4 weeks after lymphatic surgery. n=5; Data represent mean ± SD. ^*^P < 0.05 vs control; ^**^P < 0.01 vs control; ^***^P < 0.001 vs control.

Male C57BL/6J mice were anesthetized via isoflurane inhalation for surgical procedures. A 3-mm circumferential dermal excision was performed between 1.5-1.8 cm from the tail base. Subsequent lymphatic mapping was achieved through intradermal injection of 20 μl sterile 1% (w/v) Patent Blue dye (Beyotime, Y025045, China) 2-cm from the tail tip, enabling visualization of two lateral lymphatic vessels adjacent to the caudal vein. The lymphatic vessels were selectively transected using microsurgical scissors. For the sham group, the same dermal excision was performed, but the lymphatic vessels were carefully preserved and not transected.Volumetric assessment of the mouse tail was performed post-operatively by measuring its diameter of the mouse tail using the truncated cone volume formula: πh (R^2^+ Rr +r^2^)/3 (**Figure 1A**). Fifteen mice were used for this study. Five mice were analyzed for each experimental group (Control, Sham and Model), n = 5 mice were analyzed.

### 2.2 NIR-II imaging

Lymphatic drainage dynamics were evaluated through intradermal injection of 20 μl ICG at a concentration of 2.5 mg/ml (Aladdin Scientific, I107931, China) in the distal tail region, using a two-dimensional InGaAs camera (Princeton Instruments and Raptor Photonics) platform with a camera employing a variable exposure time to observe the structure of lymphatic vessels and ICG drainage in the tail.

### 2.3 HE staining

Tail tissue samples were collected four weeks post-surgery and fixed in 4% paraformaldehyde for 24 h. After paraffin embedding, 5µm-thick sections were cut and mounted on adhesive slides. Deparaffinization was performed by xylene immersion, followed by ethanol gradient hydration. The sections were processed with hematoxylin and eosin and finally processed by sealing the sections for visualization under a microscope.

### 2.4 Multiplex immunofluorescence staining of macrophage subtypes

The sections were deparaffinized in xylene, followed by rehydration in a descending series of ethanol solutions. Antigen retrieval was performed by heating the sections in citrate buffer (pH 6.0) using a microwave oven. Endogenous peroxidase activity was quenched by incubation with 3% H_2_O_2_ for 15 min, followed by blocking with 5% normal goat serum in PBS for 30 min at room temperature.

To identify M1 macrophages, a three-step sequential tyramide signal amplification (TSA) staining was applied: first with F4/80 (Tyramide-488, green), followed by CD80 (Tyramide-555, red), and finally iNOS (Tyramide-647, far-red). For M2 macrophage identification, the same TSA procedure was performed using F4/80 (Tyramide-488, green), CD206(Tyramide-555, red), and Arg1 (Tyramide-647, far-red). After the final TSA cycle, nuclear were counterstained with DAPI for 5 min at room temperature. The sections were washed twice with PBS, mounted with anti-fade mounting medium, and imaged using a confocal laser scanning microscope(Zeiss, LSM980). The macrophage ratio was expressed as the percentage of CD80^+^iNOS^+^F4/80^+^(CD206^+^Arg1^+^F4/80^+^) triple-positive cells relative to the total F4/80^+^ macrophage. Quantitative fluorescence analysis was performed using Image J software.

### 2.5 Polarised M2 macrophages and preparation of M2-CM

Human Acute Monocytic Leukemia Cells (THP-1) were cultured in RPMI-1640 supplemented with 10% fetal bovine serum. These cells were induced into M2 macrophages according to a previously established method (26). The cells were cultured in fresh RPMI-1640 basal medium for 24 h. The medium was then centrifuged to collect the supernatant, which was then labeled as M2-CM and prepared for subsequent experiments. GW4869 (Beyotime, S1971, China) was used to inhibit M2 macrophage exosome secretion. After THP-1 cells were induced to differentiate into M2 macrophages, they were cultured in fresh RPMI-1640 medium containing 20 μM GW4869 for 24 h. The medium was then centrifuged to collect the supernatant, which was labeled as M2+GW4869-CM.

### 2.6 Extraction and identification of M2-Exo

After M2 macrophage differentiation was completed, the culture supernatant was discarded, and the cells were gently washed with PBS. The medium was then replaced with RPMI-1640 supplemented with exosome-depleted fetal bovine serum, and the cells were incubated for an additional 24 hours. M2-Exo were isolated from the conditioned medium using differential ultracentrifugation. The collected medium was first centrifuged at 3, 000×g for 20 minutes at 4 °C to remove cellular debris. The supernatant was then transferred to new tubes and centrifuged at 10, 000×g for 30 minutes at 4 °C to eliminate other extracellular vesicles and large complexes. Subsequently, the resulting supernatant was subjected to ultracentrifugation at 100, 000×g for 70 minutes at 4 °C. Morphological observation was performed using transmission electron microscopy (TEM). Particle size distribution and concentration were measured by nanoparticle tracking analysis (NTA). The expression of exosomal surface-enriched proteins, including CD9 (Proteintech, 20597-1-AP), CD81 (Proteintech, 27855-1-AP) and TSG101 (Proteintech, 28283-1-AP) was detected by Western Blot, with calnexin used as a negative marker protein.The final exosome pellet was resuspended in 200 μL of PBS. The total protein concentration of the isolated M2-Exo was determined using a bicinchoninic acid assay with a standard curve. The purified M2-Exo (100 μg) were added into the hLECs cell medium for co-incubation, and were then used for subsequent experiments.

### 2.7 Culture of hLECs

The hLECs were cultured in endothelial cell culture medium ECM (Sciencell, 1001, America). The effect of M2 macrophages on hLECs was assessed by establishing the control, LPS, M0-CM, and M2-CM groups. An LPS-induced hLECs injury model was established to simulate the inflammatory microenvironment of SL and to study hLECs dysfunction (25, 27). The control group was cultured with RPMI-1640 and ECM at a 1:1 ratio. RPMI-1640 and ECM were also used in the LPS group at a 1:1 ratio with 2 μg/ml LPS. The M0-CM and M2-CM groups were cultured with different conditioned media in a 1:1 ratio with ECM and 2 μg/ml LPS. These groups were used to assess the functionality of hLECs and for subsequent experiments.

### 2.8 EDU cell proliferation assay

hLECs were treated with different conditioned media for 24 h. After incubation with EDU, the cells were fixed and permeabilized. EDU staining was performed using the Click-iT EdU-555 Cell Proliferation Detection Kit (Servicebio, G1602, China). After staining, the EDU-positive cells were detected using fluorescence microscopy at an excitation wavelength of 550 nm.

### 2.9 Scratch experiment

hLECs in the logarithmic growth phase were treated with different conditioned media for 24 h, as previously described. A straight vertical line was scratched and the scratched cells were washed away. Incubation was continued for another 24 h before imaging. Cell migration rate = (initial scratch area after 24 h)/initial scratch area.

### 2.10 Tube formation experiment

A total of 200 μL of matrix gel (BD Biosciences; 356234, USA) was added to each well of a 24-well plate. The cells were inoculated onto the matrix gel, photographed under maximum tube formation conditions, and analyzed for tube formation length using ImageJ software.

### 2.11 Intracellular ROS detection in hLECs

ROS levels in the hLECs were quantified using the fluorescent probe DCFH-DA (Beyotime, S0033S, China). Following 24 h exposure to conditioned media as described previously, cells were incubated with DCFH-DA (10μM) in the dark. After washing with PBS, the cells were examined under a fluorescence microscope or analyzed using flow cytometry (Ex/Em = 488/525 nm).

### 2.12 Mitochondrial membrane potential (ΔΨm) assessment

ΔΨm was evaluated using a JC-1 Assay Kit (Solarbio, M8650, China). The cells were then incubated with JC-1 solution (10μg/ml) in the dark for 30 min. The cells were analyzed by dual-channel fluorescence detection. JC-1 monomers (Ex/Em = 490/530 nm) and JC-1 polymers (Ex/Em = 525/590 nm) were also detected. Green fluorescence indicates a decrease in ΔΨm, whereas red fluorescence indicates normal ΔΨm (28).

### 2.13 Mitochondrial permeability transition pore opening analysis

The opening status of the mPTP was detected using the mPTP Assay Kit (Abbkine; KTA4002, China). The hLECs were incubated with Calcein AM (1 μM) and cobalt chloride (4 mM) to selectively quench Calcein AM fluorescence from cell membranes. After incubation, Calcein AM was detected (Ex/Em=494/517 nm). Greater green fluorescence indicates a lower degree of mPTP opening (29).

### 2.14 Cellular immunofluorescence

After fixation with 4% paraformaldehyde, non-specific binding was blocked using goat serum. The primary antibody against Nrf2 (Bioss, bs-1074R, China) was incubated at 4 °C overnight. After washing with PBS, the sections were incubated with a Cy3-conjugated secondary antibody (Beyotime, A0516, China), followed by nuclear staining with DAPI. Finally, the cells were observed using fluorescence microscopy (Ex/Em=550/570 nm).

### 2.15 Reverse transcription quantitative PCR

Total RNA was extracted and reverse-transcribed into cDNA (MCE, HY-K0510A, USA). Quantitative PCR amplification was performed using SYBR Green qPCR Master Mix (MCE, HY-K0501A, USA). Primer sequences used in this study are listed in **Supplementary Table 1**. Relative mRNA expression normalized to β-actin was calculated using the 2^-ΔΔCT^ method as previously described (30).

### 2.16 Western blot

Tissue and cellular proteins were extracted using RIPA buffer (Beyotime, P0013B, China). Nuclear fractions from the hLECs were extracted using a Nuclear Protein Extraction Kit (Solarbio, EX1470, China). The samples (30µg) were separated on 8% SDS-PAGE gel (Yeasen, 20324ES62, China) and wet-transferred to PVDF membranes. After blocking with 5% non-fat milk, the membranes were incubated with the following primary antibodies:Nrf2 (Bioss, bs-1074R, China), VEGFR3 (Santa Cruz, sc-514825, 1:1000), and LYVE1 (Novus Biologicals. NB600-1008, 2 µg/mL), Keap1 (Wanlei, WL03285, China), CD206 (Wanlei, WL06177, China), CD80 (Wanlei, WL02639, China), HO-1 (Wanlei, WL02400, China), NQO1 (Wanlei, WL04860, China) lamin B1 (Abways, P20700, China) and β-actin (Bioworld, P68133, China). Following overnight incubation, membranes were treated with HRP-conjugated secondary antibodies (Bioss, bs-80295G-HRP, China). Signals were tested using the BeyoECL Plus kit (Beyotime, P0018, China), and protein bands were captured using a Mini Chemi610 imaging system (SinSage Technology).

### 2.17 Statistical analysis

GraphPad Prism 8.0 software was used to analyze the data. Experiments were independently repeated three times, and data is expressed as mean ± standard deviation (mean ± SD). Additionally, statistical significance between groups was assessed using t-test or One-way ANOVA, with *P* < 0.05 considered statistically significant.

## 3 Results

### 3.1 Decreased M2 macrophage infiltration in tissues of secondary lymphedema

We established a model of SL in C57 BL/6 mice by performing tail skin excision combined with lymphatic vessel ligation. The results showed that, compared with the control group, the sham group developed mild swelling from day 5 post-surgery which subsequently subsided, whereas the model group exhibited significant edema starting from day 5 and persisting until week 4, with a 58.3 ± 10.3% increase in edema at week 4 (*P* < 0.05) (**Figures 1A, B**). ICG near-infrared fluorescence imaging (31) showed that within 30 min, ICG injected into the tail could reflux along the caudal lymphatic vessels to the sacral lymph nodes in normal mice. In the sham group, subcutaneous ICG reflux was blocked, but ICG could still reflux along the caudal lymphatic vessels to the sacral lymph nodes. In contrast, in the model mice, a large amount of ICG was retained in the distal region of the tail incision and failed to reflux to the sacral lymph nodes (**Figure 1C**). Histopathological evaluation showed that the thickness of the subcutaneous tissue in the tail was significantly increased in the model group, with obvious fat deposition (*P* < 0.05) (**Figures 1D, E**) and lymphatic vessel dilatation (shown by white arrows in **Figure 1D**), consistent with the classic pathological features of SL (31). Molecular level analysis showed that the mRNA expression of IL-1β and TNF-α was significantly upregulated in the model group(*P* < 0.05) (**Figure 1F**), suggesting that the lymphedema microenvironment could lead to a significant inflammatory cascade response. Furthermore, the expression of antioxidant molecules such as Nrf2, HO-1, and NQO1 was suppressed in the model group, while the expression of Keap1 was increased, indicating limited antioxidant capacity in lymphedema tissues (P < 0.05) (**Figures 1G, H**). However, no significant changes were observed in the sham group. It is worth noting that surgery only induced local lymphatic dysfunction without systemic effects, as evidenced by the same trend of body weight gain in the model and control mice (*P* > 0.05)(**Supplementary Figure 1A**), with no pathological changes observed in the major organs. (**Supplementary Figure 1B**).

The infiltration of M1 and M2 macrophages during the pathological process of SL was examined by immunophenotyping. Multiplex immunofluorescence staining revealed that the fluorescence signal of F4/80 was significantly enhanced in SL tissues, indicating increased macrophage infiltration. This was accompanied by markedly increased fluorescence signals of CD80 and iNOS, whereas no significant changes were observed in the signals of CD206 and Arg1. These findings suggest substantial infiltration of M1 macrophages and a significant reduction in the relative proportion of M2 macrophages in SL tissues at the 4-week time point (**Figure 1I**). Western blotting and immunohistochemistry similarly indicated a decreased proportion of M2 macrophages in SL tissues. Specifically, Western blotting quantified the protein expression of M2 markers (CD206 and Arg-1) and M1 markers (CD80 and iNOS), while immunohistochemistry was performed using CD206 for M2 and CD80 for M1 staining, both consistently showing a reduced M2/M1 ratio. (**Supplementary Figure 2**). However, at week 6, we observed increased infiltration of M2 macrophages compared with that at week 4 (**Supplementary Figure 3**). Collectively, these results demonstrate that the pathological changes during the early stage of SL progression are characterized by increased infiltration of pro-inflammatory M1 macrophages and a relative deficiency of anti-inflammatory M2 macrophages.

### 3.2 M2-CM promotes the function of hLECs and upregulates VEGFR3 expression

Based on the above pathological features, we further investigated the regulatory mechanisms of M2 macrophage paracrine components in the functions of hLECs. An inflammation model of hLECs was established by LPS (2 μg/ml) induction (**Supplementary Figure 4**). LPS stimulation exhibited a comparable capacity to H_2_O_2_ in inducing oxidative stress injury and mitochondrial homeostasis imbalance, and also exerted similar effects on suppressing the Keap1-Nrf2 antioxidant pathways in hLECs (**Supplementary Figure 5**). LPS has been widely used in studies on lymphatic dysfunction. Therefore, we chose to use LPS to simulate the inflammatory microenvironment of SL and study hLECs dysfunction (25, 27). M2 macrophages were obtained by polarizing THP1 with IL-4 and IL-13 (**Supplementary Figure 6**). Results confirmed by macrophage-conditioned medium concentration gradient optimization experiments (**Supplementary Figure 7**), the final system of conditioned M2-CM medium was mixed 1:1 with ECM for functional studies of hLECs.

**Figure 2:**
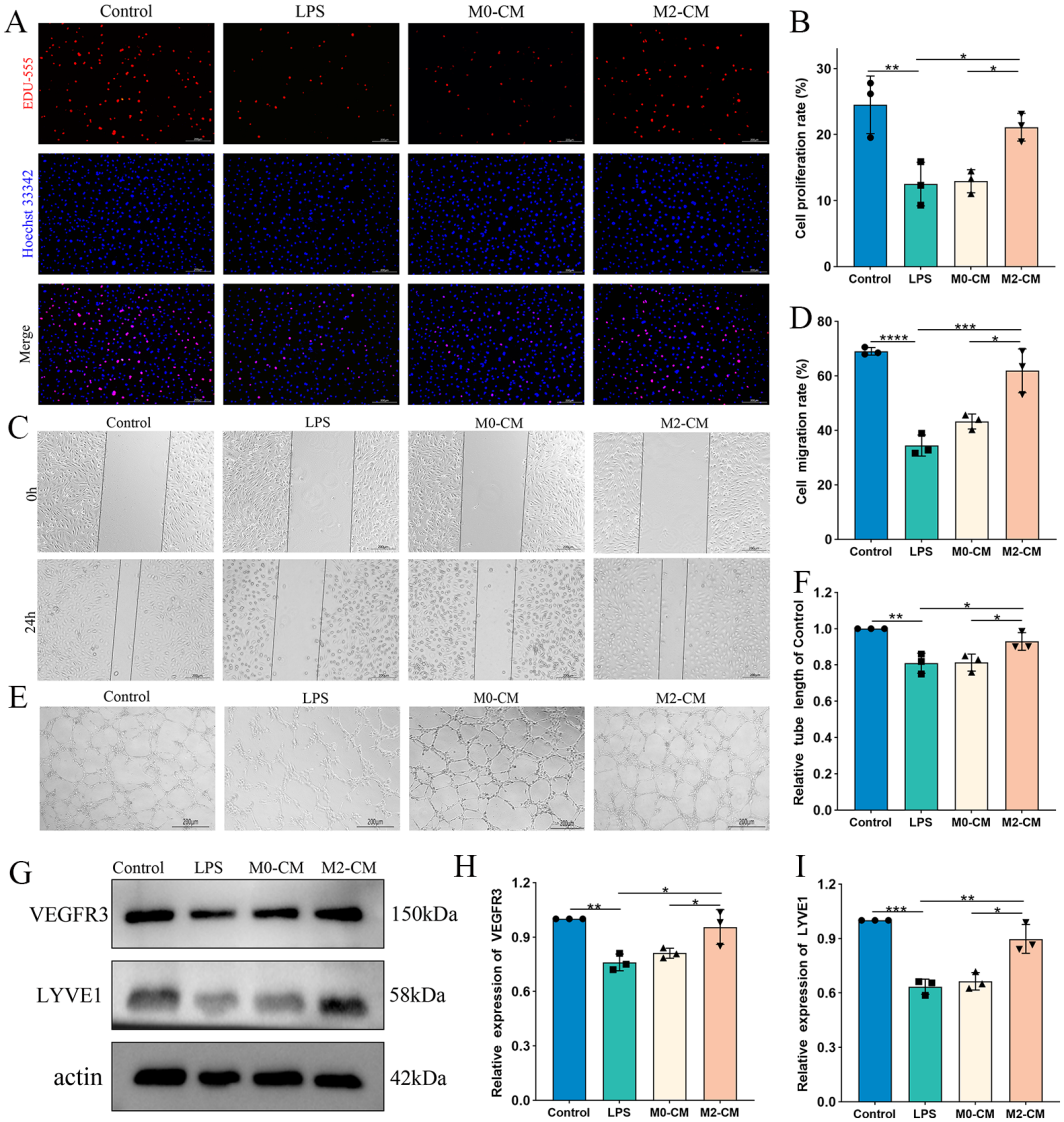
M2-CM promotes the function of hLECs and upregulates VEGFR3 expression.**(A)** EDU immunofluorescence staining of hLECs treated with M2-CM; **(B)**Statistical graphs of hLECs proliferation rate in EDU experiments; **(C)** Cell scratch assay of hLECs treated with M2-CM; **(D)** Statistical graphs of cellular migration rate of hLECs in cellular scratch assay; **(E)** Tube formation assay of hLECs treated with M2-CM; **(F)** Relative lengths of tubulogenesis represented in the statistical graph; **(G)** Representative WB images of VEGFR3/LYVE1 in hLECs treated with M2-CM; **(H)** Quantitative analysis of VEGFR3 expression; **(I)** Quantitative analysis of LYVE1 expression. Data represent the mean ± SD. ^*^P < 0.05; ^**^P < 0.01; ^***^P < 0.001.

The EDU cell proliferation assay showed that the proliferation rate of hLECs was up-regulated by 8.63 ± 1.20% after 24 h of M2-CM intervention compared with the LPS group(*P* < 0.05) (**Figures 2A, B**). The results of the cell scratch migration assay showed that the migration rate of hLECs was up-regulated by 27.44 ± 4.15% compared to the LPS group (*P* < 0.05) (**Figures 2C, D**). Three-dimensional Matrigel tube formation experiments further demonstrated that M2-CM intervention resulted in a significant increase in the length of lymphatic vessel structures (*P* < 0.05) (**Figures 2E, F**). Molecular studies showed that the LPS significantly inhibited the expression of key lymphangiogenic markers VEGFR3 and LYVE1 protein expression (*P* < 0.05) (**Figures 2G–I**), whereas M2-CM intervention significantly upregulated VEGFR3 and LYVE1 protein expression(*P* < 0.05) (**Figures 2G–I**). The above results suggest that M2-CM can prevent the inflammation-induced inhibition of hLECs function and upregulate the VEGFR3/LYVE-1 signaling axis.

### 3.3 M2-CM maintains hLECs mitochondrial homeostasis by regulating mPTP opening

**Figure 3:**
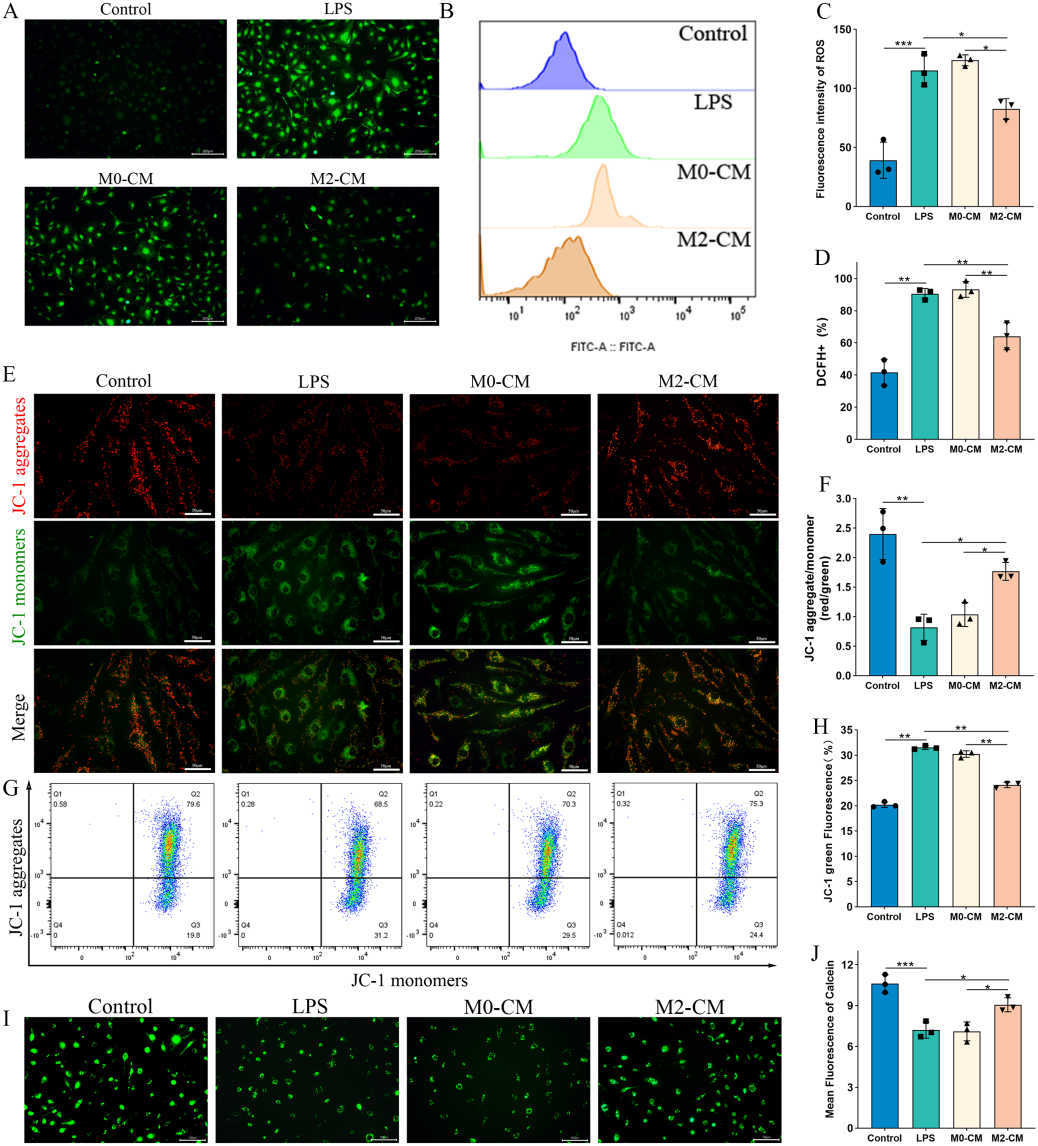
M2-CM reduces mitochondrial damage in hLECs by inhibiting the hyperactivation of mPTP.**(A)** Fluorescence of hLECs ROS detected using DCFH-DA; **(B)** Flow histogram of hLECs ROS detected using DCFH-DA; **(C)** Immunofluorescence intensity of ROS in hLECs; **(D)** Positivity statistics of DCFH in flow cytometry; **(E)** Fluorogram of ΔΨm in hLECs detected using JC-1; **(F)** Statistics on the ratio of JC-1 aggregates to monomers in hLECs; **(G)** Flow scatter plot of ΔΨm in hLECs detected using JC-1; **(H)** Statistics of the green fluorescence of JC-1 in hLECs detected by flow cytometry; **(I)** Fluorogram of mPTP in hLECs detected using Calcein AM. **(J)** Immunofluorescence intensity statistics of mPTP in hLECs detected using Calcein AM. Data represent the mean ± SD. ^*^P < 0.05; ^**^P < 0.01; ^***^P < 0.001.

Based on the above functional findings, we investigated the molecular pathways through which M2-CM regulates mitochondrial homeostasis in hLECs. qRT-PCR Quantitative analysis showed that M2-CM suppressed the mRNA expression of IL-1β, TNF-α, and IL-18 in hLECs (*P* < 0.05) (**Supplementary Figure 8**), suggesting that M2-CM could counteract the inflammatory response. Fluorescence detection by DCFH-DA probe showed that M2-CM decreased the ROS fluorescence intensity in hLECs compared with that in the LPS group (*P* < 0.05) (**Figures 3A, C**). The quantitative results of flow cytometry also showed that the proportion of DCFH+ cells in the M2-CM group was significantly lower than that in the LPS group (*P* < 0.05) (**Figures 3B, D**), whereas M0-CM intervention failed to show a significant antioxidant effect (*P* < 0.05) (**Figure 3A-D**). Normal ΔΨm is key to maintaining mitochondrial function and cellular energy metabolism, and increased ROS levels may disrupt ΔΨm, leading to mitochondrial and hLECs dysfunction (19). ΔΨm analysis showed that the JC-1 aggregates/monomer ratio in the M2-CM group was significantly increased, indicating that it was able to effectively maintain the integrity of ΔΨm in the cells of hLECs (*P* < 0.05) (**Figures 3E, F**). Quantitative analysis by flow cytometry also showed that the M2-CM group displayed higher JC-1 aggregates fluorescence and lower JC-1 monomer fluorescence (*P* < 0.05) (**Figures 3G, H**), suggesting that ΔΨm can be maintained at a higher level by M2-CM. Opening of the mPTP leads to a lower ΔΨm and increased ROS accumulation, ultimately causing oxidative stress damage to endothelial cells (32). Assessment of the mPTP opening status using calcein AM/CoCl2 staining revealed that in the LPS group, more CoCl2 entered the mitochondria, resulting in calcein fluorescence quenching, whereas the M2-CM intervention group retained more calcein fluorescence signals, which was significantly better than that of the M0-CM group (*P* < 0.05) (**Figures 3I, J**). The above experiments indicated that M2-CM could inhibit ROS upregulation and block mitochondrial damage on the one hand, while preventing excessive opening of the other hand, thus preventing the vicious cycle of oxidative stress and mitochondrial dysfunction caused by the decrease in ΔΨm.

### 3.4 M2-CM activates hLECs antioxidant defense network through Keap1-Nrf2 pathway

**Figure 4:**
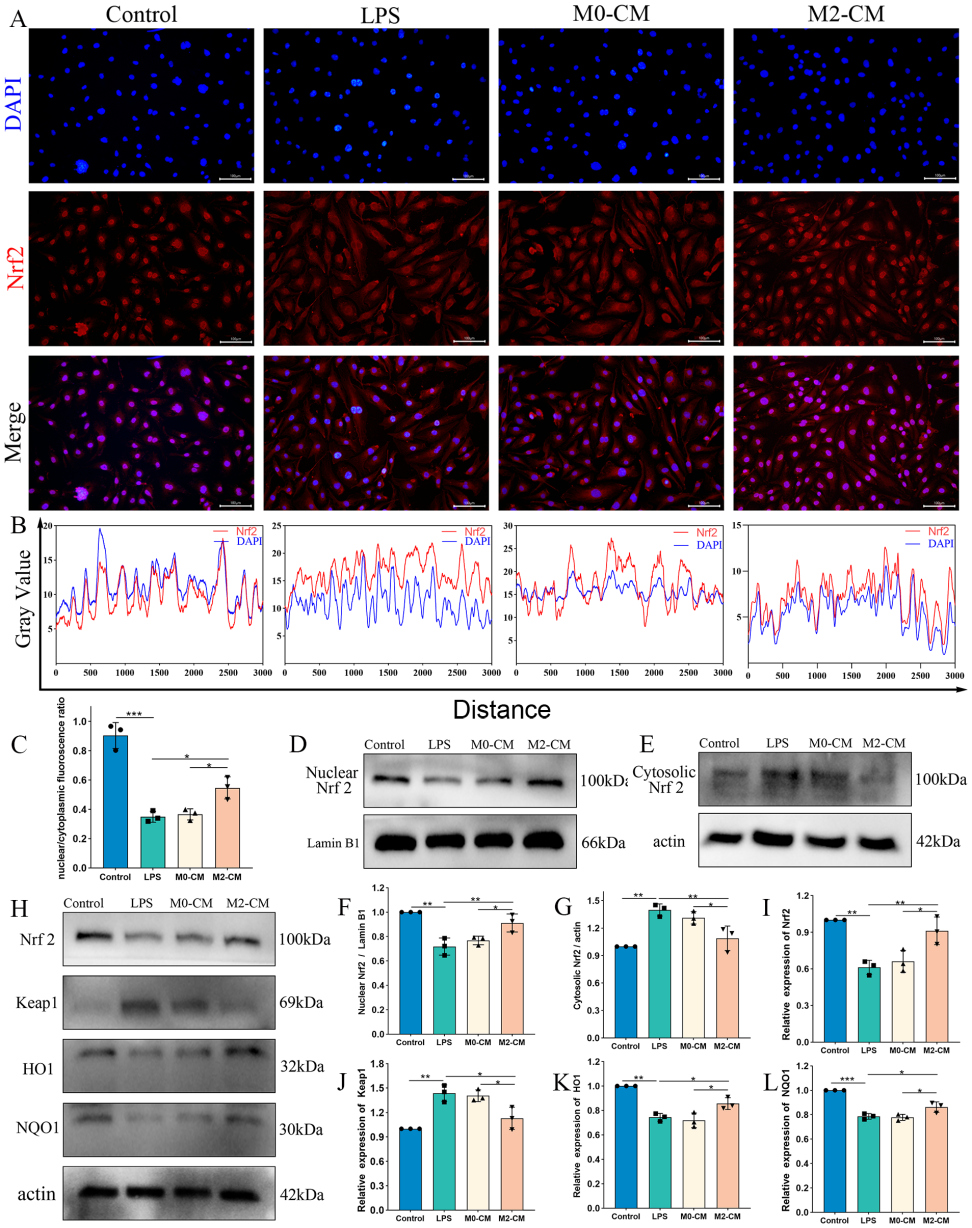
M2-CM promotes nuclear translocation of Nrf2 in hLECs. **(A)** Immunofluorescence staining of Nrf2 in hLECs treated with M2-CM; **(B)** Trend of colocalization of Nrf2 and DAPI channels in merged images; **(C)** Statistical ratio of Nrf2 signals in the nucleus to the cytoplasm; **(D)** Representative WB images of Nrf2 in the nuclear fraction; **(E)** Representative WB images of Nrf2 in the cytoplasm; **(F)** Quantitative analysis of nuclear Nrf2 expression; **(G)** Quantitative analysis of cytosolic Nrf2 expression; **(H)** Representative WB images of total Nrf2/Keap1/HO-1/NQO1 in hLECs treated with M2-CM; **(I)** Quantitative analysis of total Nrf2 expression; **(J)** Quantitative analysis of total Keap1 expression. **(K)** Quantitative analysis of HO-1 expression; **(L)** Quantitative analysis of NQO1 expression. Data represent the mean ± SD. ^*^P < 0.05; ^**^P < 0.01; ^***^P < 0.001.

Nuclear factor erythroid 2-related factor 2(Nrf2) plays an important role in combating oxidative stress by regulating the Keap1-Nrf2/ARE signaling pathway (33). Nrf2 nuclear translocation inhibits pro-inflammatory cytokines and upregulates several antioxidant gene transcripts (34). To verify the direct regulatory role of Nrf2 on mPTP, we performed intervention experiments using the Nrf2 inhibitor ML385, the Nrf2 agonist Sulforaphane, and the mPTP-specific inhibitor CsA (35–37). The results showed that the combination of LPS and ML385 further suppressed the expression of Nrf2, whereas the combination of LPS and Sulforaphane upregulated Nrf2 expression (*P* < 0.05) (**Supplementary Figures 9A, B**). More importantly, inhibition of Nrf2 by ML385 exacerbated mPTP opening and aggravated ΔΨm loss, while the addition of CsA significantly alleviated these detrimental effects(*P* < 0.05) (**Supplementary Figures 9C–F**). Activation of Nrf2 by Sulforaphane reduced mPTP opening and restored ΔΨm; the addition of CsA further improved mPTP closure and normalized ΔΨm(*P* < 0.05) (**Supplementary Figure 9C–F**). These findings demonstrate that CsA blocked the negative effects of ML385 and acted synergistically with the protective effect of Sulforaphane, confirming that Nrf2 directly regulates mPTP. Based on the elucidation of the mitochondrial protective mechanism, we further explored the molecular pathways of the M2-CM regulated antioxidant signaling pathway. Quantitative immunofluorescence analysis of the intracellular distribution of Nrf2 showed intense red fluorescence in the nucleus of hLECs in the control group, which showed a good trend of colocalization with nuclear staining (**Figures 4A, B**), whereas the LPS group showed a decreased fluorescence intensity in the nucleus and an increase in the cytoplasm (*P* < 0.05) (**Figures 4A–C**). In contrast, M2-CM significantly restored the red fluorescent signal in the nucleus and restored its tendency to co-localize with nuclear staining (*P* < 0.05) (**Figures 4A–C**). Subcellular localization analysis showed that the expression of Nrf2 in nuclear proteins was significantly increased in the M2-CM group(*P* < 0.05) (**Figures 4D, F**), whereas Nrf2 expression in cytoplasmic fractions Was significantly decreased (*P* < 0.05) (**Figures 4E, G**). Whole cell level analysis showed that total Nrf2 expression was also significantly upregulated in hLECs in the M2-CM group (*P* < 0.05) (**Figures 4H, I**), while Keap1 expression was downregulated and HO-1/NQO1 expression was upregulated (*P* < 0.05) (**Figures 4H–L**). These results suggested that M2-CM may activate the Nrf2 pathway by inhibiting Keap1 expression, promoting the translocation of Nrf2 into the nucleus and upregulating the expression of HO-1/NQO1, which may establish an oxidative stress defense network.

### 3.5 Exosome secretion is required for M2-CM to maintain mitochondrial homeostasis in hLECs

**Figure 5:**
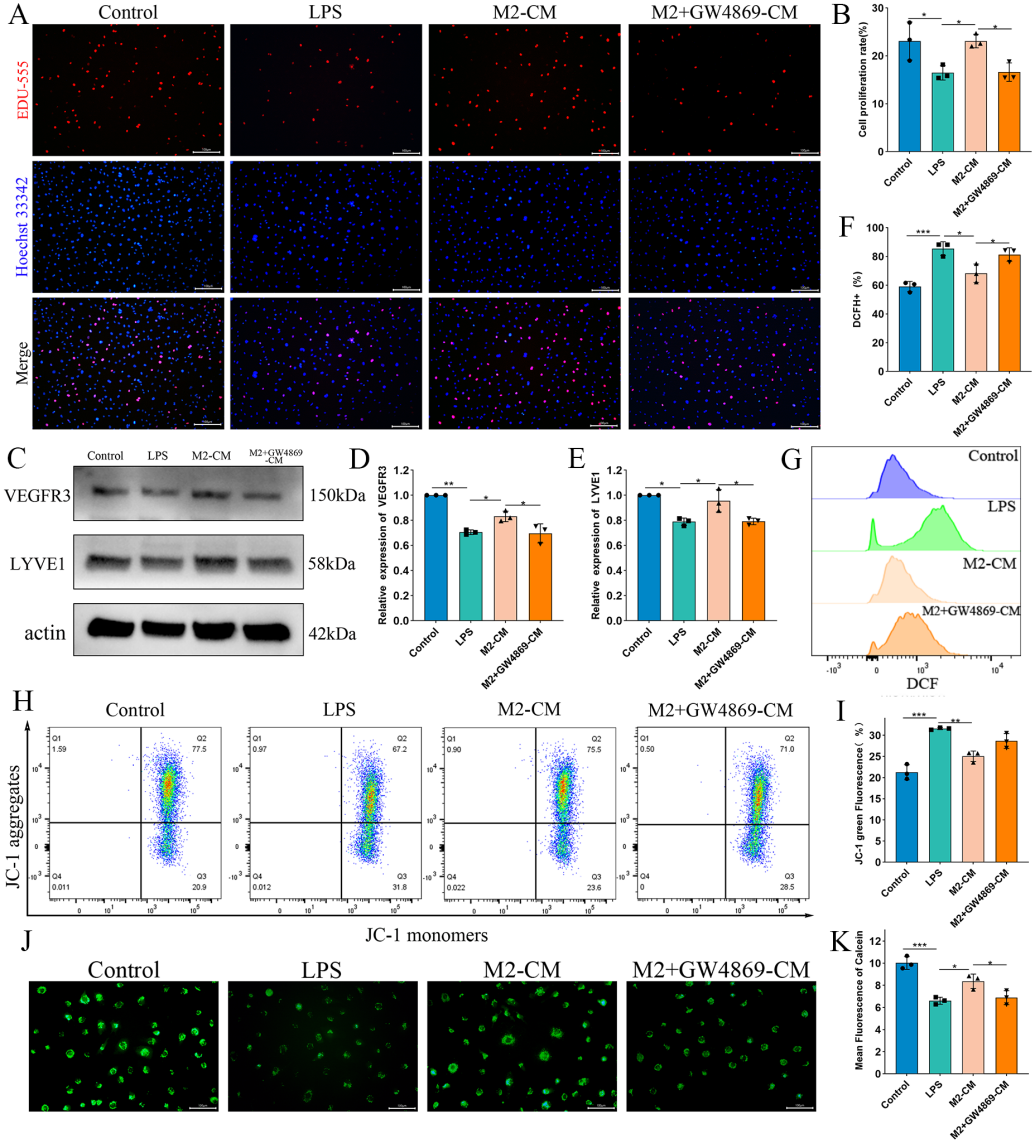
M2-CM reduces mitochondrial damage in hLECs by inhibiting mPTP over-opening, dependent on exosomes secreted by M2 macrophages. **(A)** EDU immunofluorescence staining of hLECs treated with M2+GW4869-CM; **(B)** Statistical graphs of hLECs proliferation rate in EDU experiments; **(C)** Representative WB images of VEGFR3 and LYVE1 in hLECs treated with M2+GW4869-CM; **(D)** Quantitative analysis of VEGFR3 expression; **(E)** Quantitative analysis of LYVE1 expression; **(F, G)** Flow histogram **(G)** and DCFH positivity statistics **(F)** of hLECs ROS detected using DCFH-DA; **(H)** Flow scatter plot of hLECs ΔΨm detected using JC-1; **(I)** Statistics of the green fluorescence of JC-1 in hLECs detected by flow cytometry; **(J, K)** Fluorogram **(J)** and immunofluorescence intensity statistics **(K)** of hLECs mPTP detected using Calcein AM. Data represent the mean ± SD. ^*^P < 0.05; ^**^P < 0.01; ^***^P < 0.001.

**Figure 6:**
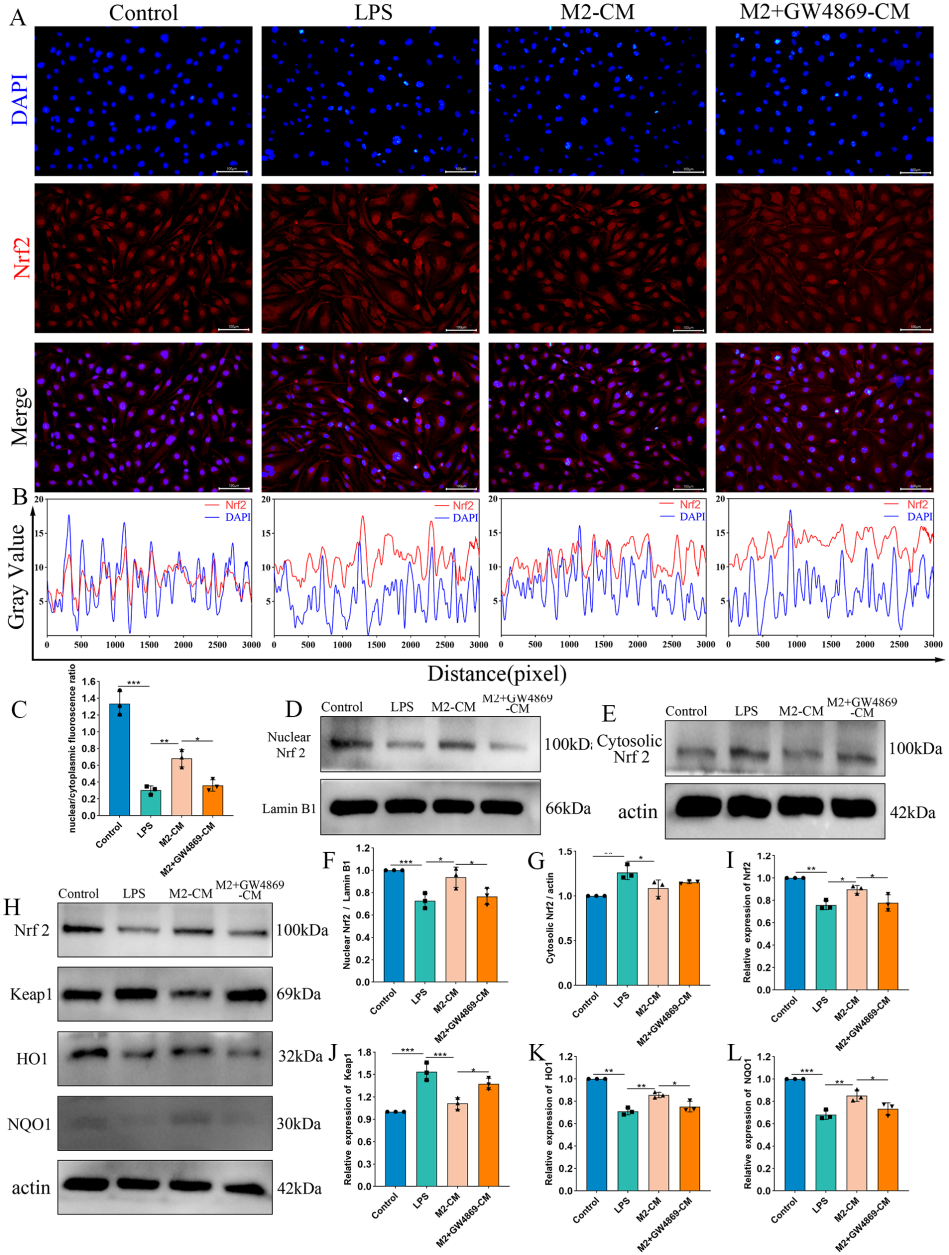
M2-CM promotes the nuclear translocation of Nrf2 in hLECs and is dependent on exosomes secreted by M2 macrophages. **(A)** Immunofluorescence staining of Nrf2 in hLECs treated with M2+GW4869-CM; **(B)** Trend of colocalization of Nrf2 and DAPI channels in merged images; **(C)** Statistical ratio of Nrf2 signals in the nucleus to the cytoplasm; **(D)** Representative WB images of Nrf2 in the nucleus; **(E)** Representative WB blots of Nrf2 in the cytoplasm; **(F)** Quantitative analysis of Nrf2 in the nucleus; **(G)** Quantitative analysis of Nrf2 in the cytoplasm; **(H)** Representative WB images of total Nrf2/Keap1/HO-1/NQO1 in hLECs treated with M2+GW4869-CM; **(I)** Quantitative analysis of total Nrf2 expression; **(J)** Quantitative analysis of total Keap1 expression; **(K)** Quantitative analysis of HO-1 expression; **(L)** Quantitative analysis of NQO1 expression. Data represent the mean ± standard deviation. ^*^P < 0.05; ^**^P < 0.01; ^***^P < 0.001.

M2-Exo can enhance anti-inflammatory effects through the biomolecules they carry (miRNAs and proteins) (38). To further clarify the regulatory role of exosomes secreted by M2 macrophages in maintaining the mitochondrial homeostasis of hLECs under M2-CM, we used GW4869 to inhibit the secretion of M2-Exo. The results showed that 20 μM GW4869 was not significantly toxic to the cell viability of hLECs and THP-1 (*P* > 0.05) (**Supplementary Figure 10**). Mechanistic studies showed that the M2+GW4869-CM resulted in a significant decrease in the pro-proliferative ability of hLECs (*P* < 0.05) (**Figures 5A, B**). Additionally, M2+GW4869-CM also led to a significantly lower expression of VEGFR3 and LYVE1 proteins (*P* < 0.05) (**Figures 5C–E**). In addition, flow cytometry results showed that M2+GW4869-CM resulted in a higher proportion of DCFH+ cells in hLECs, indicating increased oxidative stress (*P* < 0.05) (**Figures 5F, G**). Meanwhile, ΔΨm was decreased (*P* < 0.05) (**Figures 5H, I**) and mPTP opening was significantly increased (*P* < 0.05) (**Figures 5J, K**) in hLECs under M2+GW4869-CM compared with the M2-CM group. Molecular studies further revealed that exosome deficiency led to decreased Nrf2 expression and distribution in the nucleus (*P* < 0.05) (**Figures 6A–I**), while exosome deficiency led to increased Keap1 protein expression and decreased HO-1/NQO1 protein expression (*P* < 0.05) (**Figures 6H–L**). Similarly, in contrast to the M2-Exo-treated group, Exo-depleted M2-CM failed to confer effective protection on hLECs, as evidenced by impaired mitochondrial homeostasis and blunted activation of the Keap1−Nrf2 antioxidant pathway (**Supplementary Figure 11**). Taken together, these findings confirm that exosomes secreted by M2 macrophages are essential for M2-CM to protect against mitochondrial dysfunction and oxidative stress of hLECs.

### 3.6 M2-Exo promotes the function of hLECs and upregulates VEGFR3 expression

**Figure 7:**
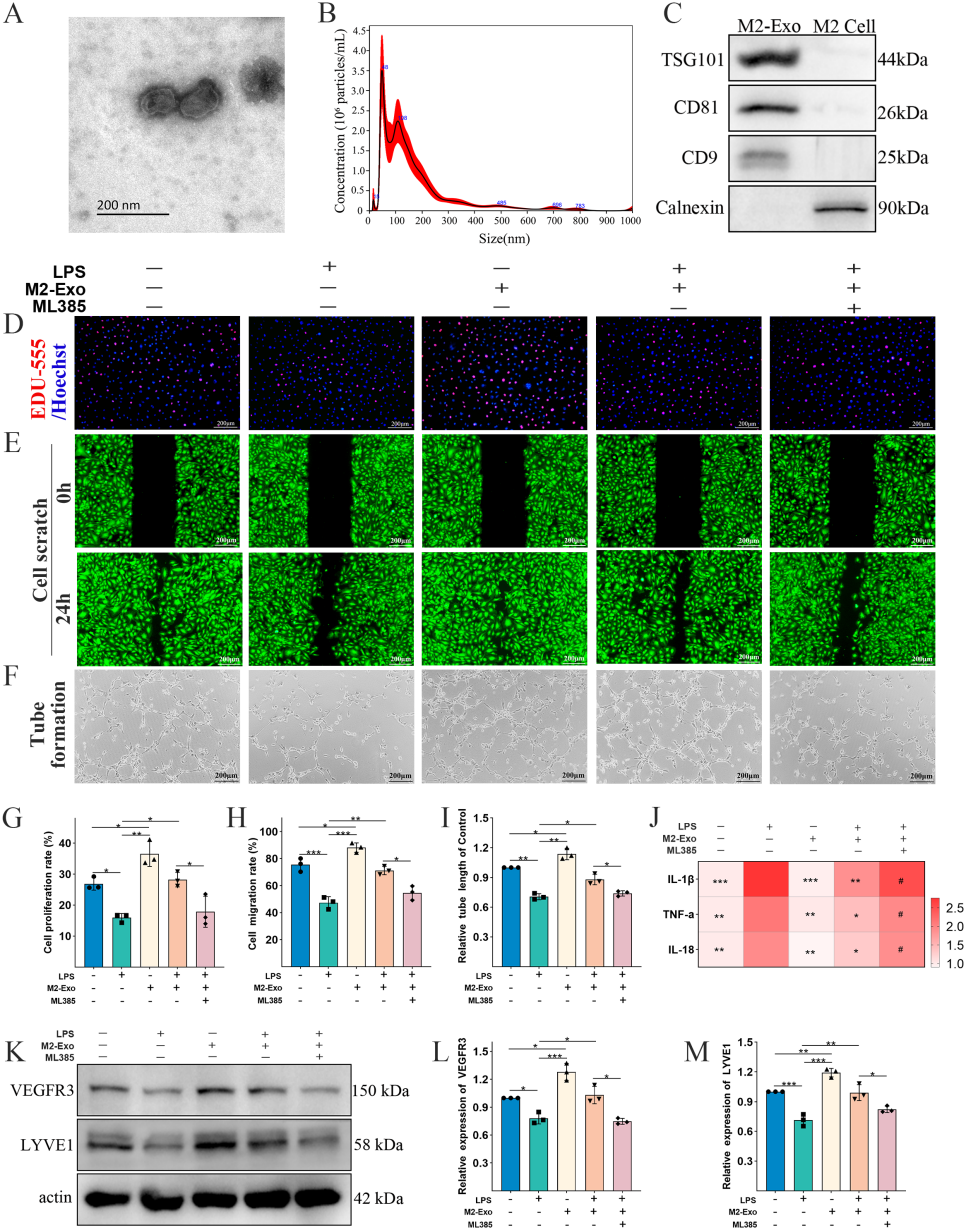
M2-Exo promotes the function of hLECs and upregulates VEGFR3 expression. **(A)** Representative electron micrograph of M2-Exo; **(B)** Nanoparticle tracking analysis (NTA) of M2-Exo; **(C)** Western blot analysis of exosome markers in M2-Exo and M2 macrophages; **(D)** EDU immunofluorescence staining of hLECs treated with M2-Exo; **(E)** Cell scratch assay of hLECs treated with M2-Exo; **(F)** Tube formation assay of hLECs treated with M2-Exo; **(G)**Statistical graphs of hLECs proliferation rate in EDU experiments; **(H)** Statistical graphs of cellular migration rate of hLECs in cellular scratch assay; **(I)** Relative lengths of tubulogenesis represented in the statistical graph; **(J)** The relative levels of the inflammatory cytokines determined by ELISA; **(K)** Representative WB images of VEGFR3/LYVE1 in hLECs treated with M2-Exo; **(L)** Quantitative analysis of VEGFR3 expression; **(M)** Quantitative analysis of LYVE1 expression. Data represent the mean ± SD. ^*^P < 0.05; ^**^P < 0.01; ^***^P < 0.001.

After dissolving the M2-Exo in PBS buffer, transmission electron microscopy (TEM) revealed that the M2-Exo exhibited a bilayer membrane structure and appeared as round or oval vesicles (**Figure 7A**). Nanoparticle tracking analysis (NTA) showed that the particle size distribution of the M2-Exo was mainly concentrated in the range of 40–150 nm (**Figure 7B**), which is consistent with the typical exosome size range. Western blot analysis confirmed that the isolated vesicles positively expressed the typical exosomal markers TSG101, CD81, and CD9, while the endoplasmic reticulum protein calnexin (a negative marker for exosomes) was not detected (**Figure 7C**). These results demonstrate that M2-Exo meeting the standard criteria were successfully isolated in this study.

To verify the direct protective effect of M2-Exo on hLECs, purified M2-Exo were directly applied to hLECs in this study. The experimental results showed that, compared with the control group, the M2-Exo alone group exhibited significantly enhanced proliferation, migration, and tube formation of hLECs(*P* < 0.05) (**Figures 7D–I**), along with markedly upregulated protein expression of VEGFR3 and LYVE1(*P* < 0.05) (**Figures 7K–M**), indicating that M2-Exo itself can promote lymphatic endothelial cell function. Compared with the LPS group, the LPS and M2-Exo co-treatment group showed significantly alleviated LPS-induced cellular dysfunction, as evidenced by promoted proliferation, migration, and tube formation(*P* < 0.05) (**Figures 7D–I**), as well as increased expression of VEGFR3 and LYVE1(*P* < 0.05) (**Figures 7K–M**). Furthermore, the expression levels of the inflammatory cytokines IL-1β, TNF-α, and IL-18 were significantly lower in the M2-Exo-treated group than in the LPS group, suggesting that M2-Exo can alleviate LPS-mediated inflammatory responses in hLECs(*P* < 0.05) (**Figure 7J**). However, upon addition of the Nrf2 inhibitor ML385, the protective effect of M2-Exo was significantly attenuated: proliferation, migration, and tube formation of hLECs were not improved, and the expression levels of VEGFR3 and LYVE1 were not upregulated. Meanwhile, the expression levels of IL-1β, TNF-α, and IL-18 were not effectively suppressed (**Figure 7**). These findings suggest that the protective effect of M2-Exo on hLECs may depend on activation of the Nrf2 pathway.

### 3.7 M2-Exo activates hLECs antioxidant defense network through Keap1-Nrf2 pathway

**Figure 8:**
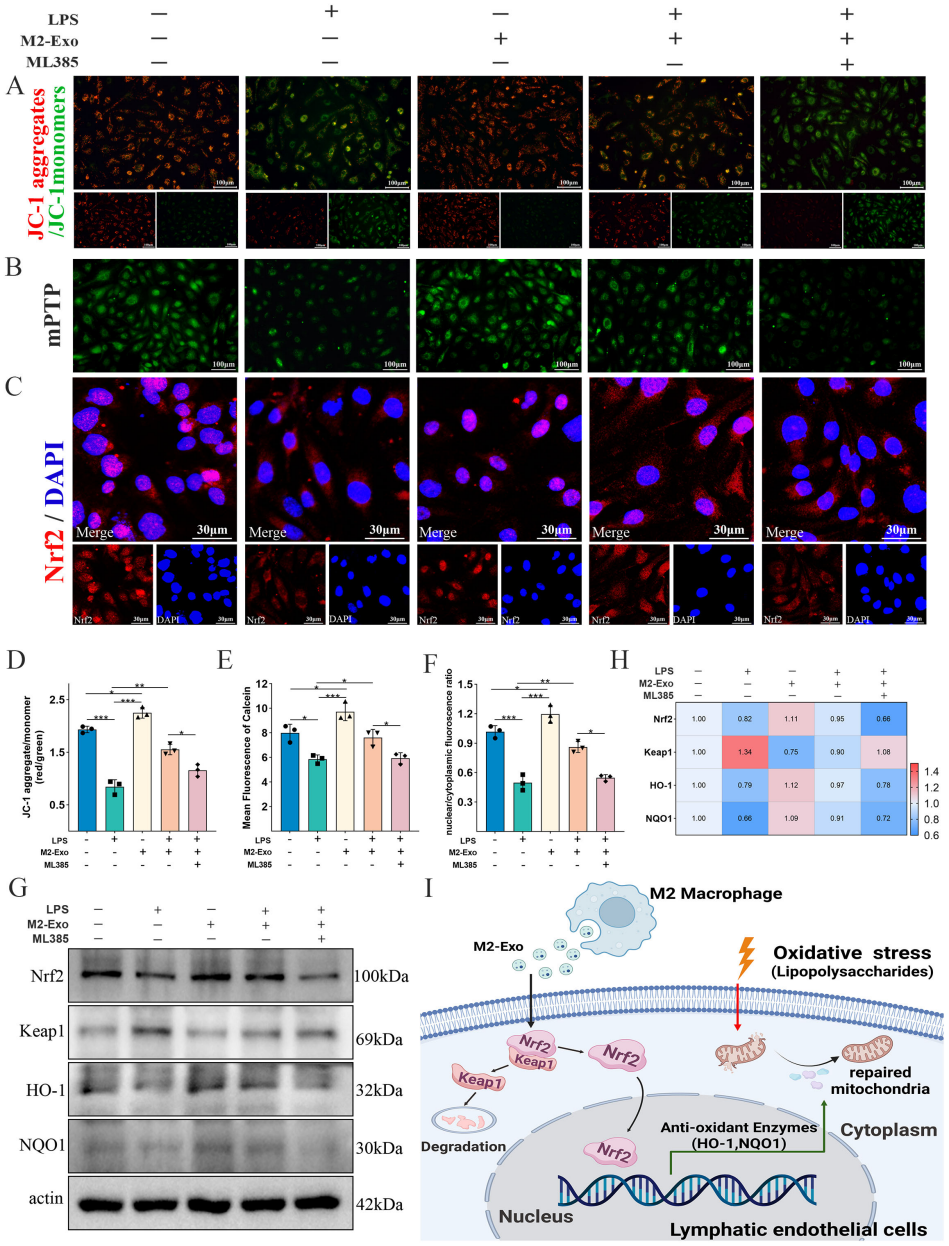
M2-Exo activates hLECs antioxidant defense network through Keap1-Nrf2 pathway. **(A)** Fluorogram of ΔΨm in hLECs detected using JC-1; **(B)** Fluorogram of mPTP in hLECs detected using Calcein AM; **(C)** Immunofluorescence staining of Nrf2 in hLECs treated with M2-Exo; **(D)** Statistics on the ratio of JC-1 aggregates to monomers in hLECs; **(E)** Immunofluorescence intensity statistics of mPTP in hLECs; **(F)** Statistical ratio of Nrf2 signals in the nucleus to the cytoplasm; **(G)** Representative WB images of total Nrf2/Keap1/HO-1/NQO1 in hLECs treated with M2-Exo; **(H)** Quantitative analysis of Nrf2/Keap1/HO-1/NQO1 expression; **(I)** M2-Exo activates Nrf2 to maintain mitochondrial homeostasis in hLECs. Data represent the mean ± SD. ^*^P < 0.05; ^**^P < 0.01; ^***^P < 0.001.

To verify whether M2-Exo activates the antioxidant defense network in hLECs through the Keap1-Nrf2 pathway, we evaluated ΔΨm, mPTP opening, Nrf2 nuclear translocation, and the expression of downstream antioxidant protein. JC-1 staining showed that the JC-1 aggregates/monomer ratio was significantly increased in the M2-Exo alone group, indicating that M2-Exo can enhance ΔΨm. Compared with the LPS group, the LPS+M2-Exo co-treatment group exhibited a significant restoration of this ratio, suggesting that M2-Exo effectively protects against LPS-induced loss of ΔΨm. However, upon addition of the Nrf2 inhibitor ML385, the protective effect of M2-Exo on ΔΨm was significantly attenuated(*P* < 0.05) (**Figures 8A, D**). Calcein AM/CoCl_2_ staining revealed that the M2-Exo alone group displayed stronger fluorescence signals, indicating closed mPTP. Compared with the LPS group, the LPS+M2-Exo co-treatment group retained significantly more calcein fluorescence, suggesting that M2-Exo inhibits aberrant mPTP opening. But after the treatment of ML385, the fluorescence signals were reduced again, indicating that the regulatory effect of M2-Exo on mPTP dependent on Nrf2 pathway(*P* < 0.05) (**Figures 8B, E**). Immunofluorescence analysis showed that the LPS+M2-Exo co-treatment group promoted Nrf2 nuclear translocation. However, ML385 treatment significantly suppressed Nrf2 nuclear translocation(*P* < 0.05) (**Figures 8C, F**). Subcellular localization analysis by Western blot also showed that M2-Exo increased the nuclear expression of Nrf2(*P* < 0.05) (**Supplementary Figure 11**). Western blot analysis revealed that the LPS+M2-Exo co-treatment group upregulated the expression of Nrf2, HO-1, and NQO1, along with suppressed the expression of Keap1. However, the addition of ML385 markedly blocked the M2-Exo-induced upregulation of Nrf2, HO-1, and NQO1(*P* < 0.05) (**Figures 8G, H**). These results demonstrate that M2-Exo can specifically inhibit the Keap1 and activate Nrf2 pathway through the exosomal delivery of functional components. This process maintains the homeostasis of Nrf2 nuclear translocation and its downstream antioxidant defence network (**Figure 8I**).

## 4 Discussion

As a common complication of lymph node dissection, the pathological process of SL is closely related to the abnormal accumulation of interstitial fluid due to impaired lymphatic fluid transport. It is clinically characterized by an inflammatory response, pathological fibrosis, and fat deposition (39). Recent research has indicated that disruption of the immune microenvironment is pivotal in the progression of SL. Specifically, the proportion of M2 macrophages varies during the course of lymphedema. In the late stage, increased M2 macrophages may contribute to pathological fibrosis (40, 41). Whereas in the early stage, insufficient M2 infiltration leads to defective anti−inflammatory and tissue−restorative functions, which may drive chronic inflammation and impaired lymphatic regeneration (17, 42). Therefore, promoting M2 polarization in the early stage represents a promising therapeutic strategy for lymphedema. Indeed, stimulation of macrophage polarization towards M2 by local injection of substance P or ultrasound-mediated piezoelectric microneedling leads to the secretion of IL-10 and IL-4. These factors inhibit inflammatory responses, promote lymphangiogenesis and reduce fibrosis to ameliorate lymphedema (18, 25). In the present study, using the mouse tail SL model, we demonstrated that lymphedema tissues showed a significant decrease in M2 macrophage infiltration at 4 weeks post-operatively, which was significantly associated with the upregulation of IL-1β and abnormality of lymphatic vessel structure. Notably, M2-Exo alleviate lymphatic vessel regenerative capacity by promoting the proliferation, migration, and tube-forming ability of hLECs, as well as upregulating the expression of VEGFR3 and LYVE1 in hLECs. These findings provide direct experimental evidence to support the development of cell-free therapeutic strategies based on the M2-Exo.

The reperfusion period following localised hypoxia in SL tissues leads to excessive oxidative stress and lipid peroxidation (14, 16). It has been shown that when mPTP is open, the free entry and exit of ions (H^+^, Ca²^+^) and small molecules into and out of the mitochondria leads to a decrease in ΔΨm, which further affects mitochondrial energy metabolism function and generates a large amount of ROS (32). In the present study, we found that M2-Exo could efficiently prevent mitochondrial dysfunction in hLECs. In the pathological microenvironment of SL, oxidative stress induced by an inflammatory response triggers the pathological opening of the mPTP, leading to a decrease in ΔΨm and burst production of ROS. Using calcein AM/CoCl2 staining, we demonstrated that M2-CM treatment significantly reduced mPTP opening and maintained ΔΨm at normal physiological levels. Notably, these effects could be blocked by the exosome secretion inhibitor GW4869, which resulted in an increase in mPTP opening, and a loss of the ability to maintain mitochondrial homeostasis. These results suggested that M2-Exo are central mediators in the regulation of mitochondrial homeostasis. Further experiments showed that M2-Exo can significantly alleviate LPS-induced functional impairment, block the vicious cycle of “ROS storm-mPTP opening” and maintains the normal physiological range of ΔΨm. These data directly demonstrate that M2-Exo mediate the protective effects on hLECs.

As a key regulator of redox homeostasis, the mechanism by which Nrf2 coordinates the cellular antioxidant defense network through the Keap1-Nrf2/ARE signaling axis has been widely reported in various diseases (43). Increased Nrf2 activity has therapeutic implications in a wide range of diseases (44–52). For example, in lung injury and inflammatory diseases, activation of the Nrf2 inhibits activation of inflammatory pathways, such as NF-κB, thereby attenuating inflammatory injury (46, 47). In neurodegenerative diseases, novel small molecules with CNS activity, such as derivatives of halogenated vinyl sulfones, can attenuate inflammatory responses by activating the Nrf2 pathway in BV-2 microglial cells, with therapeutic implications in neurodegenerative diseases (44, 48). In the peripheral vasculature, promoting Nrf2 nuclear translocation increases the expression of the downstream antioxidant protein HO-1, which reduces ROS levels, inhibits NLRP3 inflammatory vesicle formation, significantly improves cell viability and cell membrane integrity, prevents endothelial cell pyroptosis, and attenuates the progression of atherosclerosis (49, 50). Furthermore, the activation of Nrf2 nuclear translocation reduces high glucose-induced ROS production in vascular endothelial cells and endothelial damage, contributing to the regulation of impaired angiogenesis in a hyperglycemic environment. Infiltration of M2 macrophages was able to resist oxidative stress through activation of the Nrf2-HO-1 pathway, and significantly resisted H_2_O_2_-induced cell death (52). In this study, we showed that M2-Exo can inhibit Keap1 expression and upregulate Nrf2/HO-1/NQO1 expression in hLECs. We further validated the essential role of the Nrf2 pathway in the protective effect of M2-Exo using the specific Nrf2 inhibitor ML385. The results showed that ML385 effectively inhibited Nrf2 nuclear translocation and the expression of the downstream antioxidant proteins HO-1 and NQO1, and also blocked the M2-Exo mediated improvement of hLECs function and mitochondrial homeostasis. These findings demonstrate that the Nrf2 pathway is required for M2-Exo to counteract LPS-induced mitochondrial damage. Our results demonstrated that targeting Nrf2 may be a novel intervention approach for SL treatment from a molecular-cellular-functional perspective.

Transient opening of the mPTP can be used as an “alarm signal” to activate autophagy and other processes to remove damaged mitochondria. However, pathological opening of the mPTP leads to overloading of the mitochondrial quality control mechanism and triggers cell death. Its pathological opening has been shown to be associated with the development of various diseases (53–56). In diabetes and metabolic diseases, excessive opening of the mPTP disrupted ΔΨm, increased ROS production, impaired oxidative phosphorylation, and consequent release of pro-apoptotic proteins from mitochondria, ultimately leading to cell death (32, 53). Studies on atherosclerosis have shown that ox-LDL stimulation causes the release of mtDNA through mPTP channels into the cytoplasmic lysate, which activates the cGAS-STING pathway and leads to an inflammatory cascade (54). More importantly, opening of the mPTP was associated with dysfunction of mitochondrial respiratory chain complex I. This dysfunction leads to mitochondrial swelling, loss of membrane potential, and disturbances in energy metabolism (55). In particular, impaired mitochondrial function leads to blockage of the fatty acid β-oxidation process, which impairs the tricarboxylic acid (TCA) cycle of hLECs, thereby disrupting the metabolic state and functions of hLECs, such as proliferation and migration, which in turn affects the normal function of hLECs, leading to insufficient lymphatic vessel production or abnormal function, thus exacerbating lymphedema (56). In this study, mPTP was detected in hLECs for the first time, and it was shown that preventing excessive opening of mPTP in hLECs could maintain ΔΨm and improve hLECs function, providing direct experimental evidence for a therapeutic strategy targeting mPTP in SL.

The complex mechanisms of SL pathogenesis have led to slow progress in molecularly targeted therapies, and existing clinical interventions can only delay disease progression and alleviate symptoms but do not address the etiology of the disease (10, 11). In recent years, M2 macrophage exosomes, as a natural biological delivery system, have been shown to have a unique therapeutic value in trans-tissue repair. In infectious diseases, M2 macrophage exosomes significantly attenuate sepsis-associated acute lung injury and reduce mortality in mice by inhibiting neutrophil migration and neutrophil extracellular trap formation (38). In spinal cord injury, M2 macrophage exosomes activate the HIF-1α/VEGF signaling pathway, promote angiogenesis and neurogenesis, and enhance endothelial cell proliferation, migration, and tube formation, thereby reducing tissue damage and accelerating functional recovery (57). Moreover, M2 macrophage exosomes can also be used as drug carriers to efficiently penetrate the blood-brain barrier, inhibit neuroinflammation, and significantly improve motor function. It can inhibit neuroinflammation and significantly improve motor function (58). In terms of angiogenesis, M2 macrophage exosomes can directly stimulate the function of vascular endothelial cells by upregulating the expression of HIF-1α and VEGF, thereby enhancing angiogenesis and the microenvironment by synergistically exerting anti-inflammatory and anti-apoptotic effects to promote tissue repair (59, 60). The present study showed that M2-Exo played a significant role in alleviating LPS-mediated injury of hLECs and effectively maintained ΔΨm by regulating the opening of the mPTP. This cell-free therapy not only avoids immune rejection caused by conventional cellular therapies but also provides a technological guarantee of precise drug delivery with nano-sized particles, which is of great clinical significance (58).

However, the limitations of this study are the resolution of the functional components of exosomes and the translational applications of exosomes. Prior research has indicated that M2 exosomes are rich in nucleic acids, proteins, lipids, and miRNAs, of which only some functional components have been verified (61). In the future, we need to analyze the active components by multi-omics sequencing. In the future, we need to analyze the active components by multi-omics sequencing. In addition, although LPS was used in this study to mimic the inflammatory and oxidative stress responses in lymphedema, we acknowledge that LPS treatment can only partially recapitulate the complex inflammatory and oxidative microenvironment of lymphedema tissues, and thus cannot fully simulate the *in vivo* pathological milieu. At the mechanistic level, this study did not investigate LPS-induced damage such as apoptosis, pyroptosis, or autophagy in hLECs, nor the potential rescue effect of M2-Exo. The mechanism by which key effector molecules regulate mPTP also should be clarified, and the functional role of VEGFR3 should be further clarified. The lymphatic targeting efficiency of exosomes should be demonstrated through animal experiments. Ultimately, these improvements will facilitate translational development and clinical application of M2-Exo.

## 5 Conclusion

This study elucidates the molecular mechanism by which M2-Exo regulates the mitochondrial homeostasis of hLECs for the treatment of SL through delivering bioactive components. At the functional level, M2-Exo can promote lymphatic remodeling and activate the VEGFR3/LYVE-1 signaling axis, while at the molecular level, the activation of Nrf2 regulates the open state of mPTP, effectively inhibits ROS generation and loss of ΔΨm. This finding provides a new therapeutic target for the treatment of SL. Although the specific active molecules carried by exosomes have not been fully elucidated, it is clear that the mPTP pathway, based on the Keap1-Nrf2 signaling axis, is a key regulatory target for SL treatment. These findings provide a theoretical basis for targeted treatment of lymphatic vessel dysfunction. Moreover, they open a new direction for the development of non-cell-dependent regenerative medicine technologies, thereby offering a promising non-surgical therapeutic strategy to complement lymphovenous anastomosis in the clinical management of secondary lymphedema.

## Data Availability

The datasets presented in this study can be found in online repositories. The names of the repository/repositories and accession number(s) can be found in the article/**Supplementary Material**.

## Supplementary material

Supplementary Figure 1 Assessment of general health status in mice with SL.**(A)** Line graph of body weight changes in mice after surgery; **(B)** H&E staining of different organs in mice 4 weeks after surgery.

Supplementary Figure 2 Reduced M2 macrophage infiltration in tissues of secondary lymphedema. **(A)** Representative WB images in the tail tissues at 4 weeks after lymphatic surgery; **(B)** Quantitative analysis of protein expression; **(C)** Immunohistochemical staining of CD80/CD206 in mouse tail tissues 4 weeks after lymphatic surgery. Data represent mean ± SD. ^*^*P* < 0.05 vs control.

Supplementary Figure 3 Macrophage infiltration at 6 weeks after surgery. **(A)** Percentage of macrophages in mouse tail tissues; **(B)** Multiplex immunofluorescence staining of macrophage in mouse tail tissues 6 weeks after lymphatic surgery.

Supplementary Figure 4 Construction of an LPS-induced *In Vitro* Inflammation Model for hLECs. **(A–C)** mRNA Expression of TNF-α **(A)**, IL-1β **(B)**, and IL-18 **(C)** after LPS Treatment of hLECs. *p<0.05 vs 0 g/ml.

Supplementary Figure 5 LPS and H_2_O_2_ both induce oxidative stress and suppress the Keap1-Nrf2 antioxidant pathway in hLECs. **(A)** EDU immunofluorescence staining of hLECs; **(B)**Statistical graphs of hLECs proliferation rate in EDU experiments; **(C)** Fluorescence of hLECs ROS detected using DCFH-DA; **(D)** Immunofluorescence intensity of ROS in hLECs; **(E)** Fluorogram of ΔΨm in hLECs detected using JC-1; **(F)** Statistics on the ratio of JC-1 aggregates to monomers in hLECs; **(G)** Representative WB images of total Nrf2/Keap1/HO-1/NQO1 in hLECs; **(H)** Quantitative analysis of Nrf2/Keap1/HO-1/NQO1 expression; Data represent the mean ± SD. ^*^*P* < 0.05; ^**^*P* < 0.01; ^***^*P* < 0.001.

Supplementary Figure 6 Induction of M2 macrophages. **(A–F)** Relative mRNA expression of M1/M2 markers:IL-1β **(A)**, TNF-α **(B)**, Dectin1 **(C)**, IL-12 **(D)**, CD80 **(E)**, CD206 **(F)** in THP-1 after IL-4 and IL-13 treatments; **(G)** CD80/CD206 in THP1 after IL-4 and IL-13 treatments representative WB images; **(H)** Quantitative analysis of CD80 protein expression; **(I)** Quantitative analysis of CD206 protein expression.*p<0.05 vs M0; # p<0.05.

Supplementary Figure 7 Effect of different ratios of conditioned medium on cell viability of hLECs *P<0.05 vs Control.

Supplementary Figure 8 M2-CM inhibited LPS-induced expression of inflammatory factors in hLECs. **(A-C)** mRNA expression of TNF-α **(A)**, IL-1β **(B)**, and IL-18 **(B)** after M2-CM treatment of hLECs. *p<0.05 vs LPS.

Supplementary Figure 9 Nrf2 regulates mPTP opening and ΔΨm in LPS-stimulated hLECs. **(A)** Representative WB images of Nrf2 in hLECs; **(B)** Quantitative analysis of Nrf2 expression; **(C)** Immunofluorescence intensity statistics of mPTP in hLECs; **(D)** Statistics on the ratio of JC-1 aggregates to monomers in hLECs; **(E)** Fluorogram of mPTP in hLECs detected using Calcein AM; **(F)** Fluorogram of ΔΨm in hLECs detected using JC-1; ^*^*P* < 0.05; ^**^*P* < 0.01; ^***^*P* < 0.001.

Supplementary Figure 10 Detection of cytotoxicity of GW4869 on hLECs and THP-1 using CCK8. **(A)** Cell viability of hLECs after GW4869 treatment; **(B)** Cell viability of hLECs after GW4869 treatment.

Supplementary Figure 11 Exosome secretion is required for M2-CM to maintain mitochondrial homeostasis in hLECs. **(A)** EDU immunofluorescence staining of hLECs; **(B)** Statistical graphs of hLECs proliferation rate in EDU experiments; **(C)** Fluorescence of hLECs ROS detected using DCFH-DA; **(D)** Immunofluorescence intensity of ROS in hLECs; **(E)** Fluorogram of ΔΨm in hLECs detected using JC-1; **(F)** Statistics on the ratio of JC-1 aggregates to monomers in hLECs; **(G)** Representative WB images of total Nrf2/Keap1/HO-1/NQO1 in hLECs; **(H)** Quantitative analysis of Nrf2/Keap1/HO-1/NQO1 expression; Data represent the mean ± SD. ^*^*P* < 0.05; ^**^*P* < 0.01; ^***^*P* < 0.001.

Supplementary Figure 12 M2-Exo promotes nuclear translocation of Nrf2 in hLECs. **(A)** Representative WB images of Nrf2 in the nuclear and cytoplasm; **(B)** Quantitative analysis of nuclear Nrf2 expression; **(C)** Quantitative analysis of cytosolic Nrf2 expression; ^*^*P* < 0.05; ^**^*P* < 0.01; ^***^*P* < 0.001.
