# Supplementary material for: M2 macrophage−derived exosomes improves secondary lymphedema through cellular mitochondrial homeostasis regulation via the Keap1-Nrf2/mPTP axis
Source: Front Immunol. 2026 Jul 10;17:1832261. doi: 10.3389/fimmu.2026.1832261 (PMC13395612; doi:10.3389/fimmu.2026.1832261)
Supplement: Supplementary Figure 1 — Assessment of general health status in mice with SL.(A) Line graph of body weight changes in mice after surgery; (B) H&E staining of different organs in mice 4 weeks after surgery. [file SupplementaryFile1.doc]

**M2-CM improves secondary lymphedema through cellular mitochondrial homeostasis regulation via the Keap1-Nrf2/mPTP axis**

Jinli Ma^1,2,3†^, Luya Pu^1,2,3†^, Yundong Zhang^1,2,3^, Baiao Wu^1,2,3^, Rui Fei^1,2,3^, Dongmei Han^1,2,3^, Miao Hao^*1,2,3^, Jianshi Du^*1,2,3^

^1^ China-Japan Union Hospital of Jilin University, Changchun, China;

^2^ Jilin Provincial International Joint Research Center for Lymphatic Vascular Disease, Key Laboratory of Lymphatic Surgery of Jilin Province, Changchun, China

^3^ Engineering Laboratory of Lymphatic Surgery of Jilin Province, Changchun, China

^†^ These authors contributed equally: Jinli Ma, Luya Pu

✉ Miao Hao

[miaohao@jlu.edu.cn](mailto:miaohao@jlu.edu.cn)

✉Jianshi Du

[dujs@jlu.edu.cn](mailto:dujs@jlu.edu.cn)

**1. Supplementary Table S1**

**2. Supplementary Figure S1-S12**

Table S1 The primer sequences used for RT-qPCR.

| Genes | Forward primer（5’—3’） | Reverse primer（5’—3’） |
| --- | --- | --- |
| IL-1β（mouse） | GAAATGCCACCTTTTGACAGTG | TGGATGCTCTCATCAGGACAG |
| TNF-α（mouse） | CCCTCACACTCACAAACCAC | ACAAGGTACAACCCATCGGC |
| IL-18（mouse） | CCTTTGAGGCATCCAGGACA | GGGAACAGCCAGTGTTCAGT |
| β-actin（mouse） | GGAATCCTGTGGCATCCATGA | GTCTTTACGGATGTCAACGTCACAC |
| IL-1β（human） | ATGATGGCTTATTACAGTGGCAA | GTCGGAGATTCGTAGCTGGA |
| TNF-α（human） | CCTCTCTCTAATCAGCCCTCTG | GAGGACCTGGGAGTAGATGAG |
| IL-12（human） | CTCTGGCAAAACCCTGACC | GCTTAGAACCTCGCCTCCTT |
| CD80（human） | GGAGGCAGGGAACATCACCATC | AAAGACCAGCCAGCACCAAGAG |
| Dectin1（human） | AACCACAGCTACCCAAGAAAAC | GGGCACACTACACAGTTGGTC |
| CD206（human） | ACCTCACAAGTATCCACACCATCG | GGGTCCCATCACTCCACTCAAAG |
| IL-18（human） | TCTTCATTGACCAAGGAAATCGG | TCCGGGGTGCATTATCTCTAC |
| β-actin（human） | CACCATTGGCAATGAGCGGTTC | AGGTCTTTGCGGATGTCCACGT |


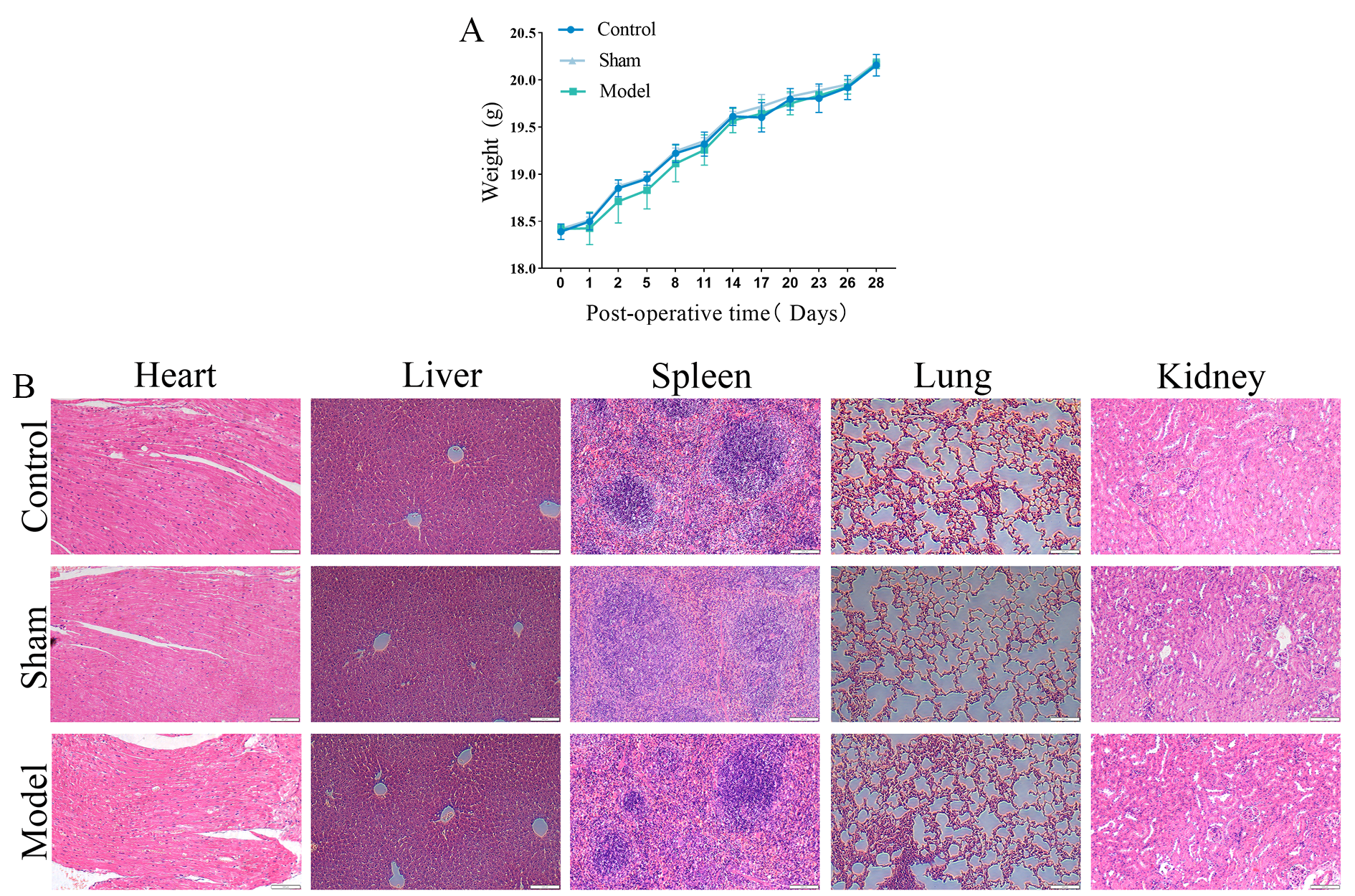


FIGURE S1

Assessment of general health status in mice with SL.**(A)** Line graph of body weight changes in mice after surgery; **(B)** H&E staining of different organs in mice 4 weeks after surgery.


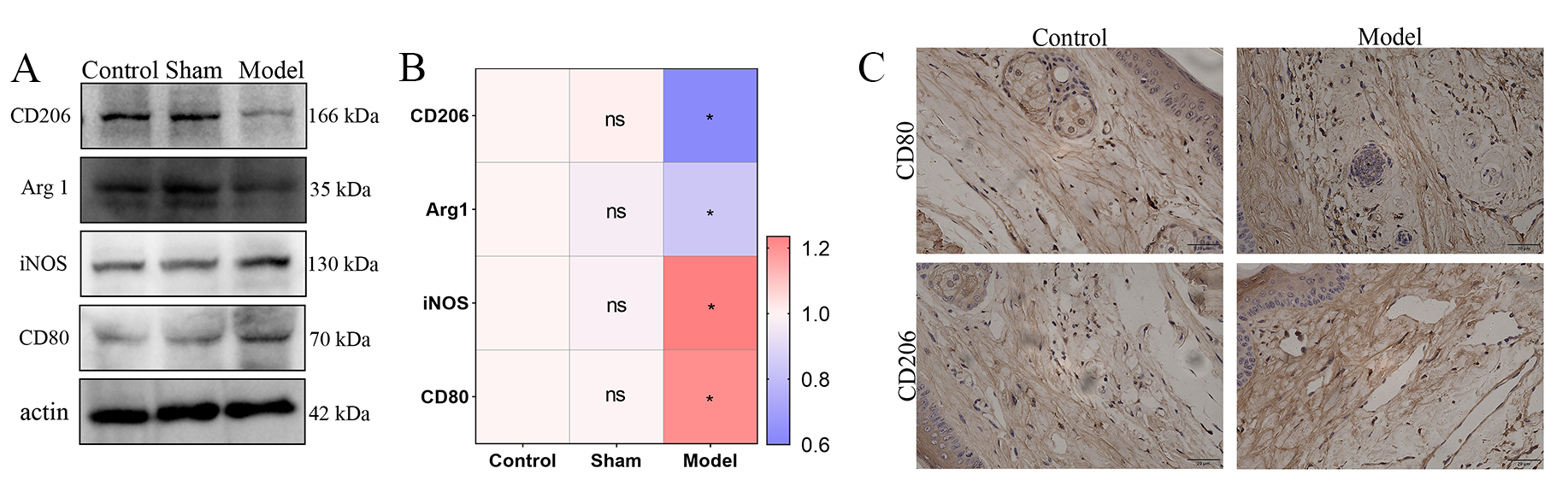


FIGURE S2

Reduced M2 macrophage infiltration in tissues of secondary lymphedema.

**(A)**Representative WB images in the tail tissues at 4 weeks after lymphatic surgery; **(B)**Quantitative analysis of protein expression; **(C)** Immunohistochemical staining of CD80/CD206 in mouse tail tissues 4 weeks after lymphatic surgery. Data represent mean ± SD. ^＊^*P*<0.05 vs control.


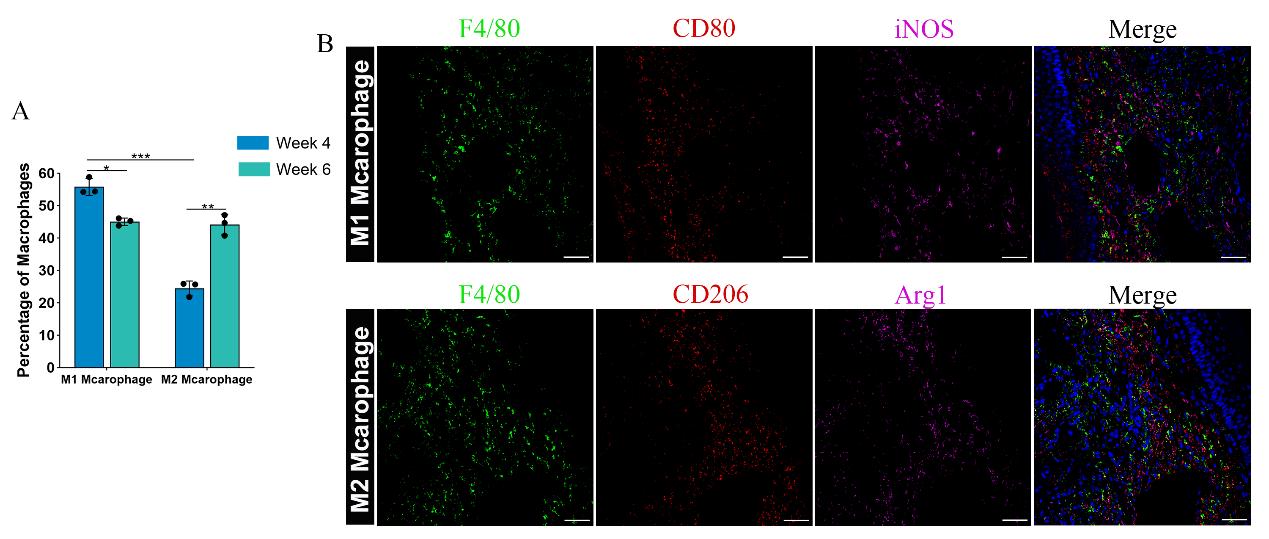


FIGURE S3

Macrophage infiltration at 6 weeks after surgery.**(A)** Percentage of macrophages in mouse tail tissues；**（B）** Multiplex immunofluorescence staining of macrophage in mouse tail tissues 6 weeks after lymphatic surgery.


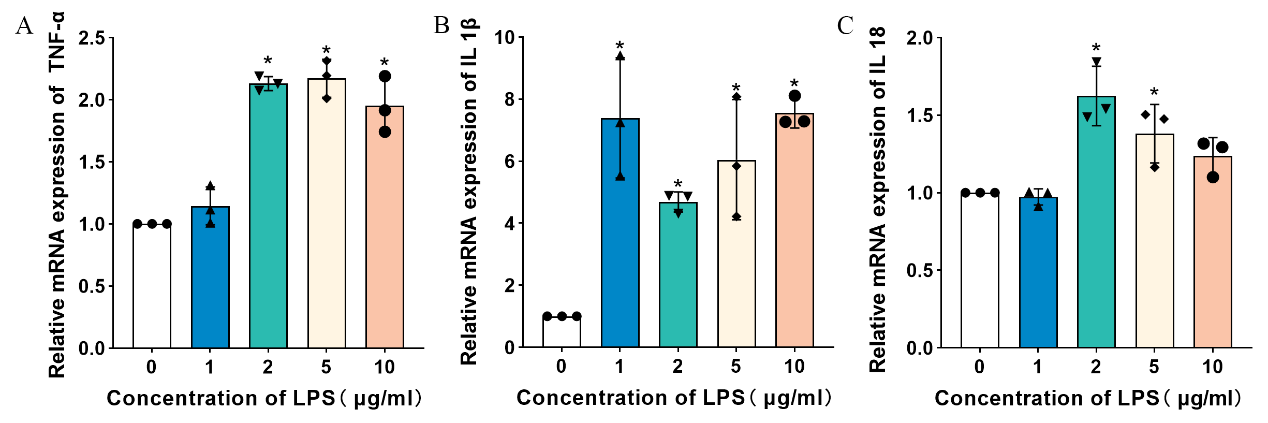


FIGURE S4

Construction of an In Vitro Inflammation Model for hLECs. **(A-C)**mRNA Expression of TNF-α(A), IL-1β(B), and IL-18(C) after LPS Treatment of hLECs. *p<0.05 vs 0 g/ml


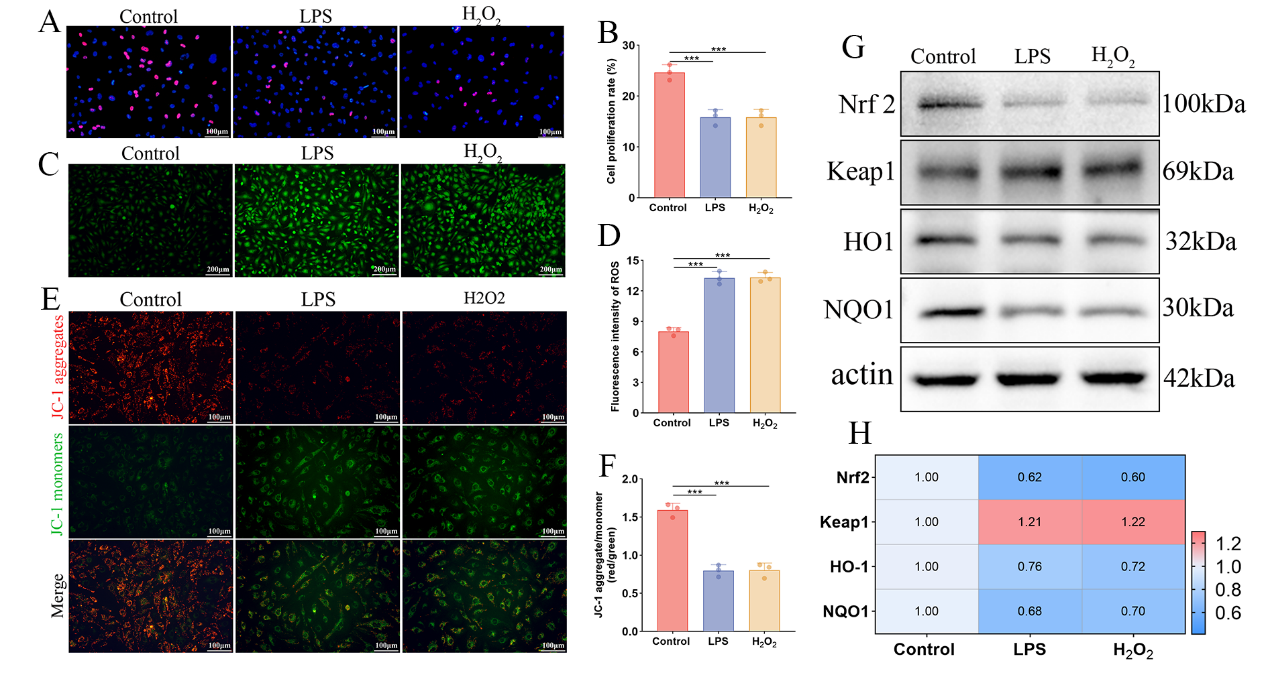


FIGURE S5

LPS and H₂O₂ both induce oxidative stress and suppress the Keap1‑Nrf2 antioxidant pathway in hLECs. **(A)** EDU immunofluorescence staining of hLECs; **(B)**Statistical graphs of hLECs proliferation rate in EDU experiments; **(C)** Fluorescence of hLECs ROS detected using DCFH-DA; **(D)** Immunofluorescence intensity of ROS in hLECs; **(E)** Fluorogram of ΔΨm in hLECs detected using JC-1; **(F)** Statistics on the ratio of JC-1 aggregates to monomers in hLECs; **(G)** Representative WB images of total Nrf2/Keap1/HO-1/NQO1 in hLECs; **(H)** Quantitative analysis of Nrf2/Keap1/HO-1/NQO1 expression; Data represent the mean ± SD. ^＊^*P*<0.05; ^＊＊^*P*<0.01; ^＊＊＊^*P*<0.001.


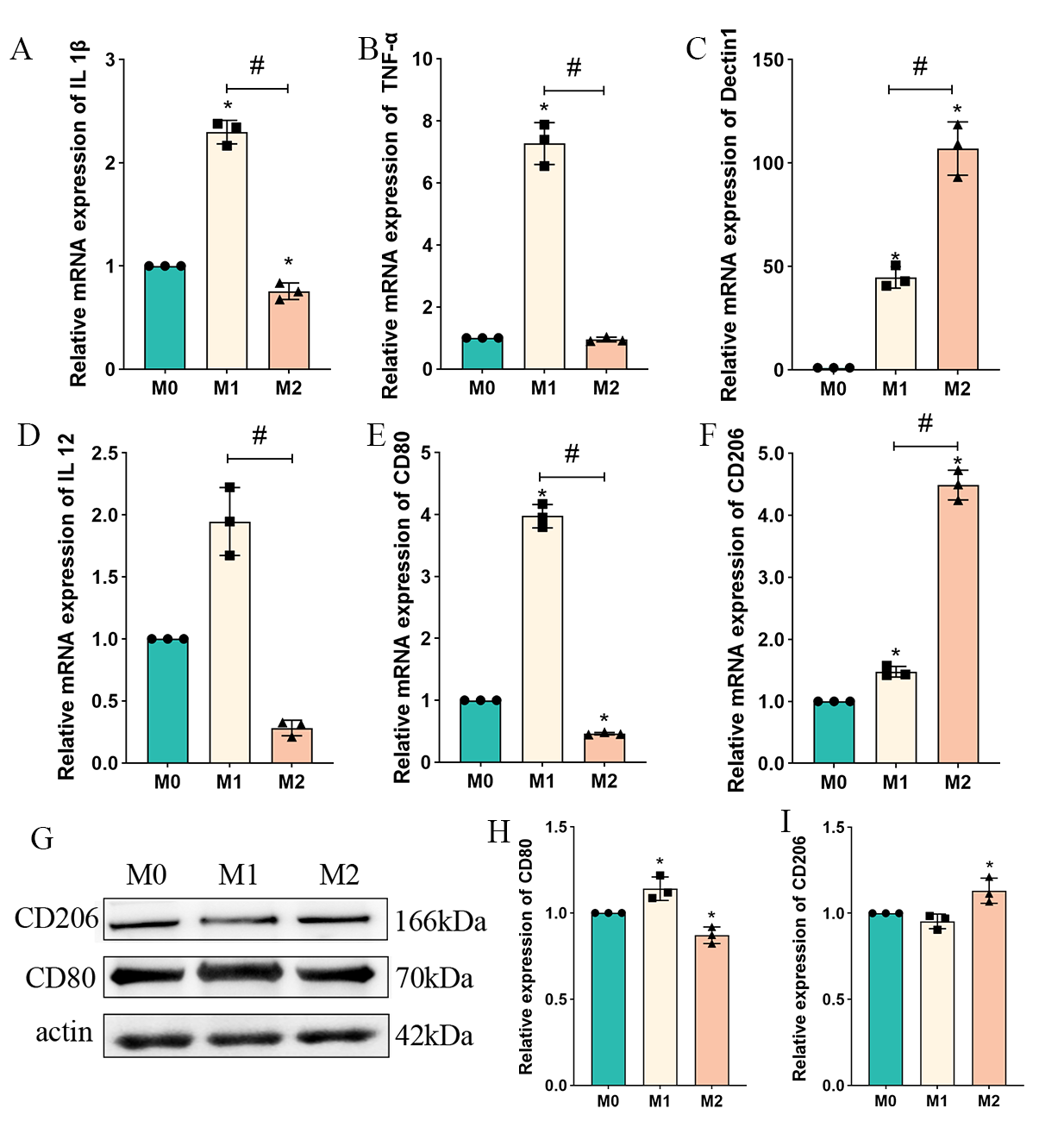


FIGURE S6

Induction of M2 macrophages. **(A-F)** Relative mRNA expression of M1/M2 markers:IL-1β (A), TNF-α (B), Dectin1 (C), IL-12 (D), CD80 (E), CD206 (F) in THP-1 after IL-4 and IL-13 treatments; **(G)** CD80/CD206 in THP1 after IL-4 and IL-13 treatments representative WB images; **(H)** Quantitative analysis of CD80 protein expression; **(I)** Quantitative analysis of CD206 protein expression.*p<0.05 vs M0； # p<0.05.


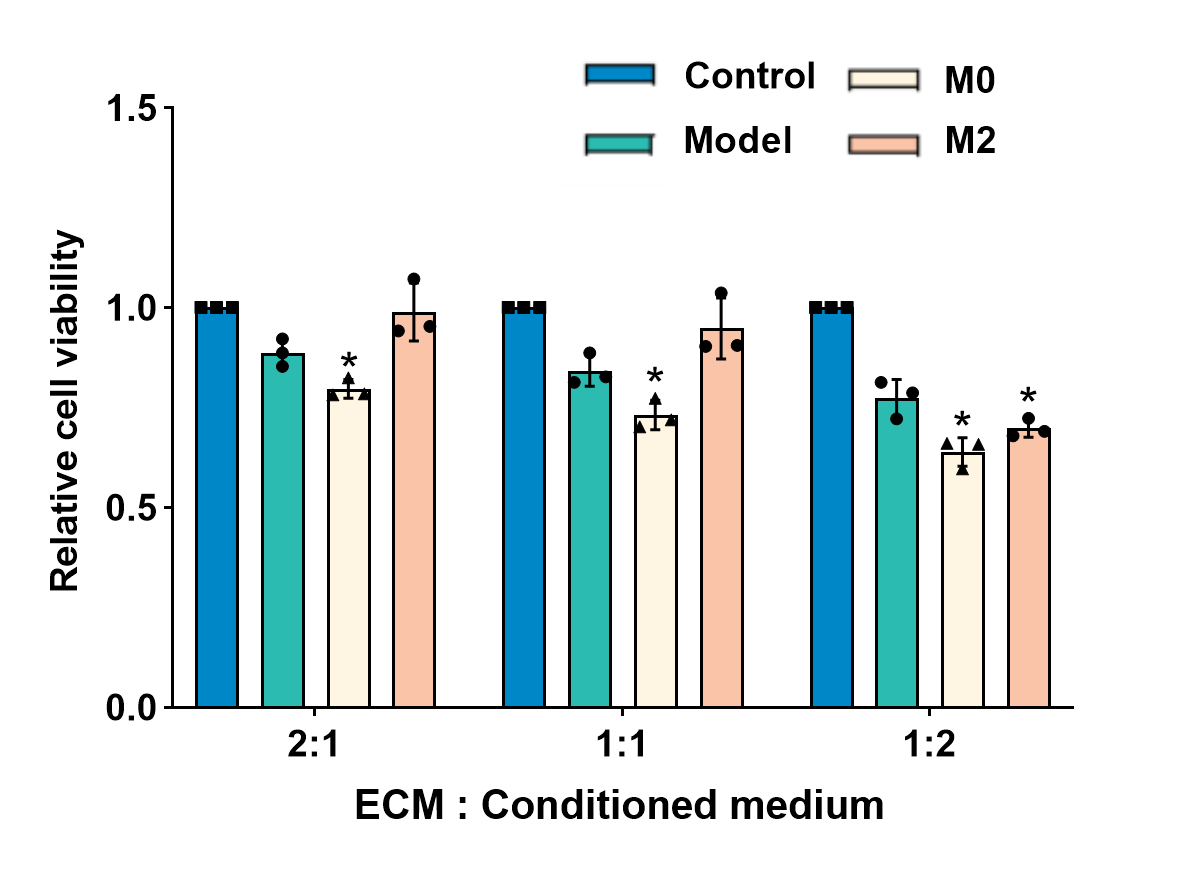


FIGURE S7

Effect of different ratios of conditioned medium on cell viability of hLECs * P＜0.05 vs Control


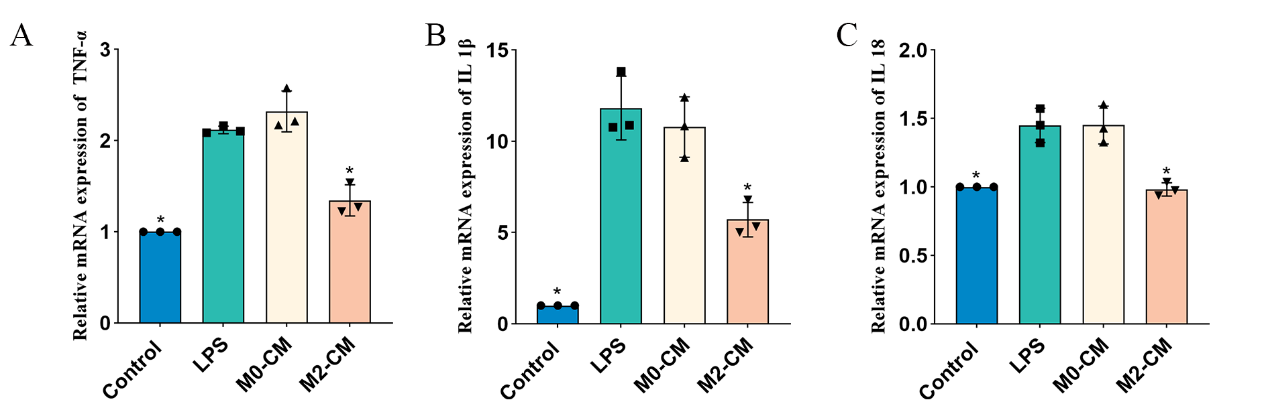


FIGURE S8

M2-CM inhibited LPS-induced expression of inflammatory factors in hLECs. **(A-C)** mRNA expression of TNF-α(A), IL-1β(B), and IL-18(B) after M2-CM treatment of hLECs. *p<0.05 vs LPS


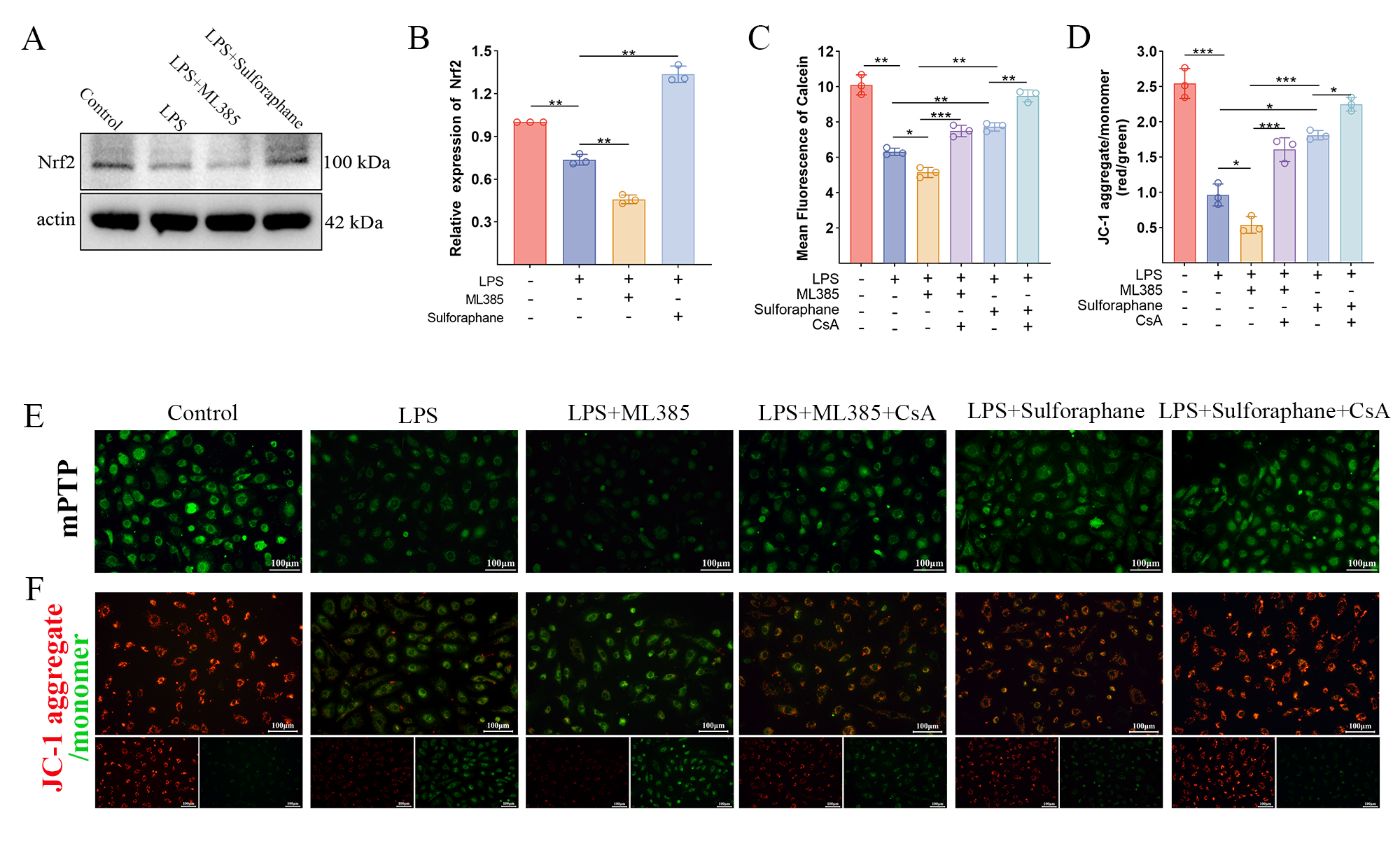


FIGURE S9

Nrf2 regulates mPTP opening and ΔΨm in LPS-stimulated hLECs. **(A)** Representative WB images of Nrf2 in hLECs; **(B)** Quantitative analysis of Nrf2 expression; **(C)** Immunofluorescence intensity statistics of mPTP in hLECs; **(D)** Statistics on the ratio of JC-1 aggregates to monomers in hLECs;  **(E)** Fluorogram of mPTP in hLECs detected using Calcein AM; **(F)** Fluorogram of ΔΨm in hLECs detected using JC-1; ^＊^*P*<0.05; ^＊＊^*P*<0.01; ^＊＊＊^*P*<0.001.


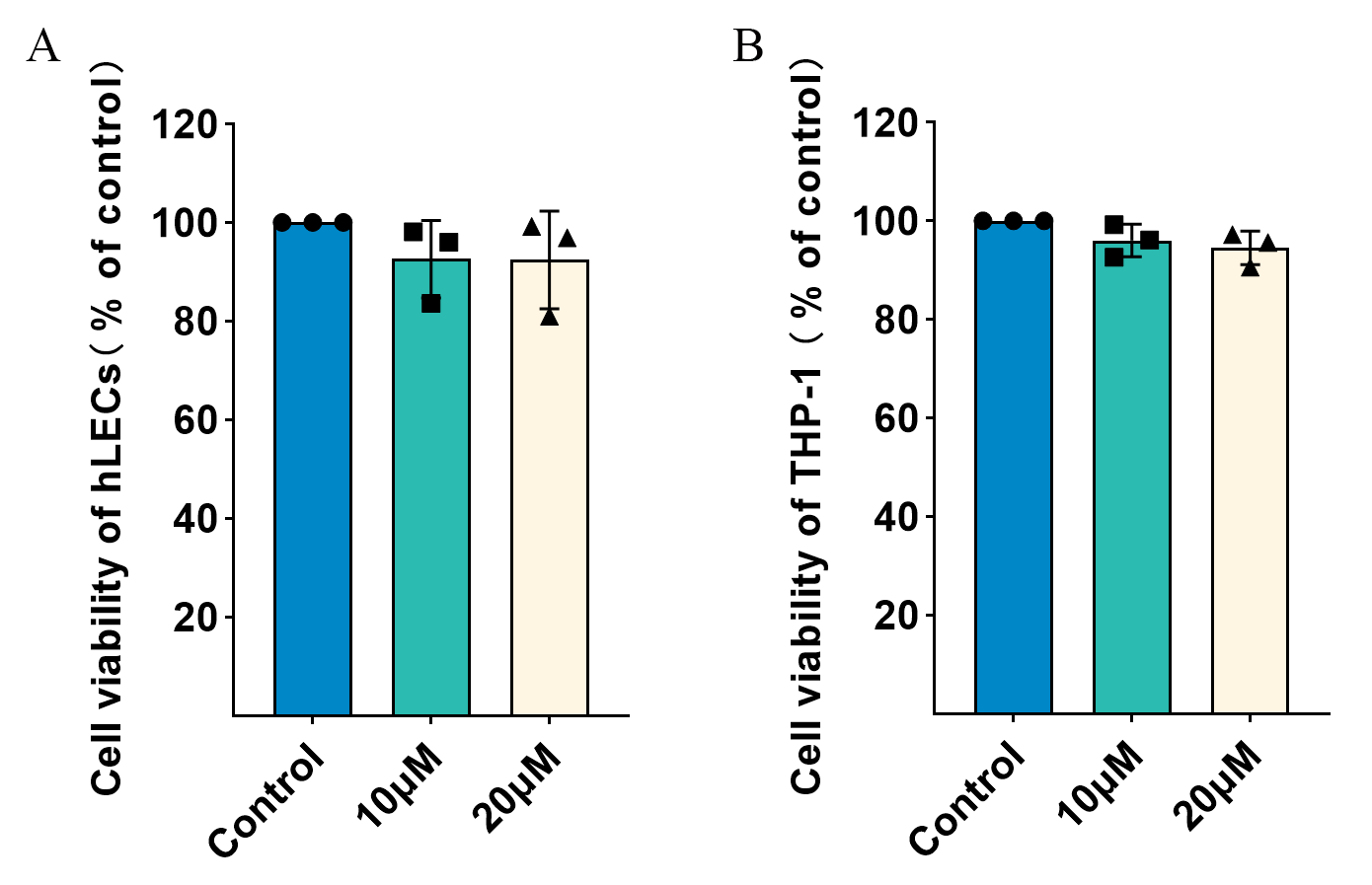


FIGURE S10

Detection of cytotoxicity of GW4869 on hLECs and THP-1 using CCK8. **(A)**Cell viability of hLECs after GW4869 treatment; **(B)**Cell viability of hLECs after GW4869 treatment


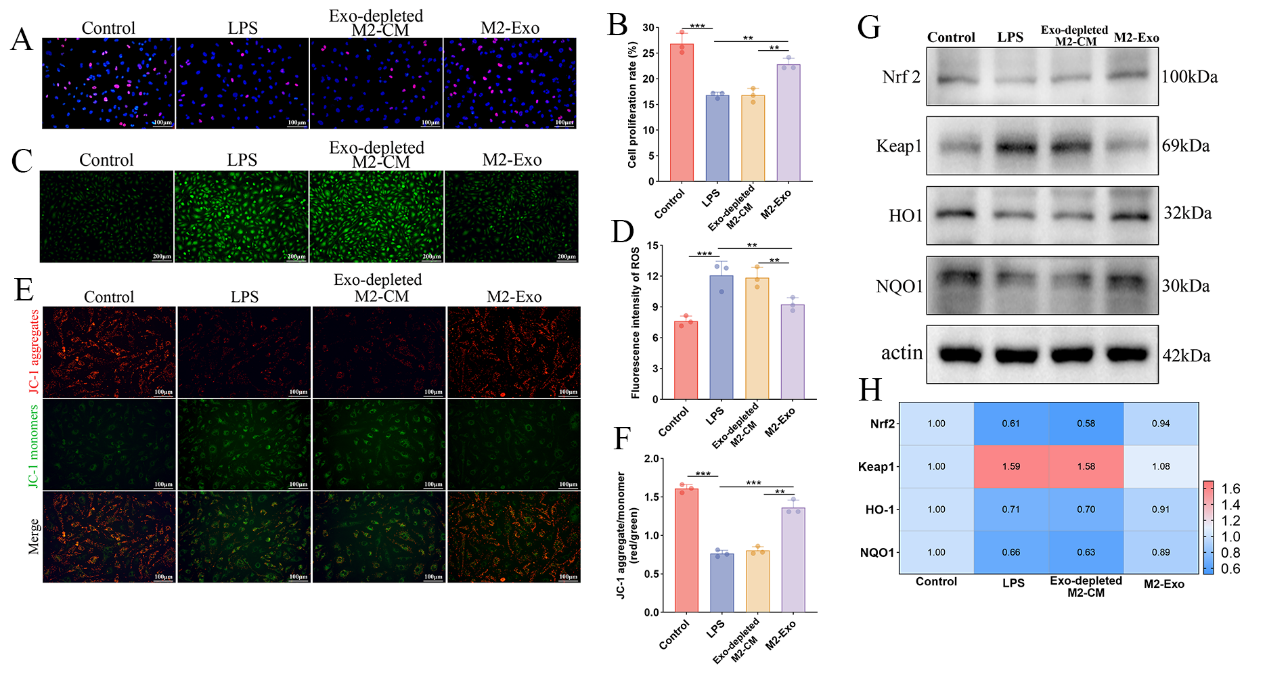


FIGURE S11

Exosome secretion is required for M2-CM to maintain mitochondrial homeostasis in hLECs. **(A)** EDU immunofluorescence staining of hLECs; **(B)**Statistical graphs of hLECs proliferation rate in EDU experiments; **(C)** Fluorescence of hLECs ROS detected using DCFH-DA; **(D)** Immunofluorescence intensity of ROS in hLECs; **(E)** Fluorogram of ΔΨm in hLECs detected using JC-1; **(F)** Statistics on the ratio of JC-1 aggregates to monomers in hLECs; **(G)** Representative WB images of total Nrf2/Keap1/HO-1/NQO1 in hLECs; **(H)** Quantitative analysis of Nrf2/Keap1/HO-1/NQO1 expression; Data represent the mean ± SD. ^＊^*P*<0.05; ^＊＊^*P*<0.01; ^＊＊＊^*P*<0.001.


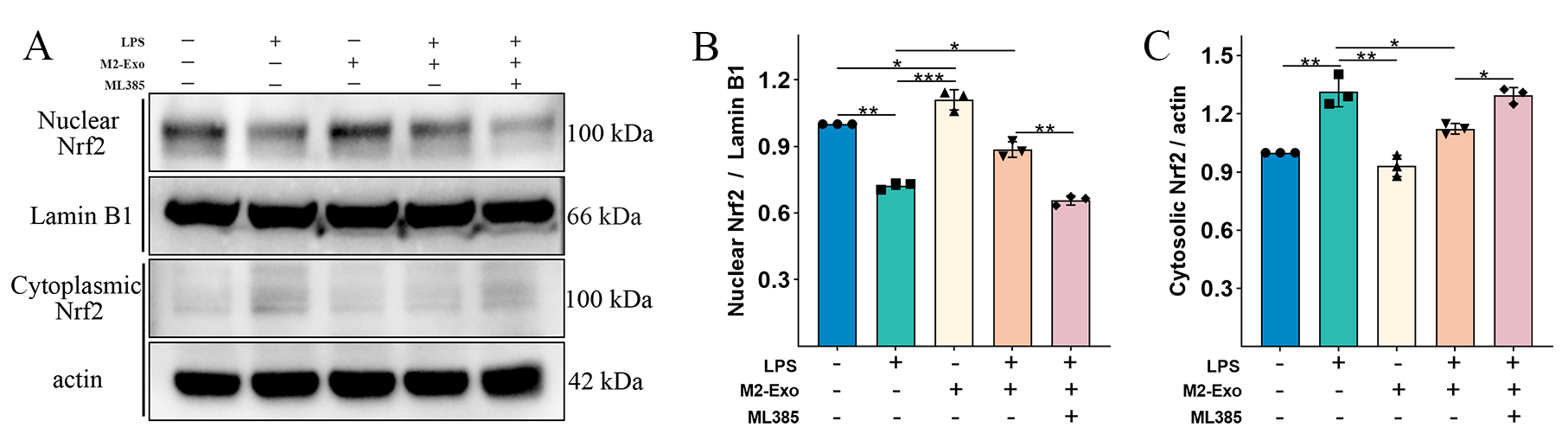


FIGURE S12

M2-Exo promotes nuclear translocation of Nrf2 in hLECs . **(A)** Representative WB images of Nrf2 in the nuclear and cytoplasm; **(B)** Quantitative analysis of nuclear Nrf2 expression; **(C)** Quantitative analysis of cytosolic Nrf2 expression; ^＊^*P*<0.05; ^＊＊^*P*<0.01; ^＊＊＊^*P*<0.001.
